# Shock transmissions and business linkages among US sectors

**DOI:** 10.1007/s10479-022-04979-8

**Published:** 2022-12-05

**Authors:** Linh Xuan Diep Nguyen, Thanaset Chevapatrakul, Simona Mateut

**Affiliations:** 1 School of Business, University of Leicester, Brookfield, London Road, Leicester, LE2 1RQ UK; 2 Business School, University of Nottingham, Jubilee Campus, Nottingham, NG8 1BB UK

**Keywords:** Asset pricing, Stock market, Shock transmission, Variance decomposition, Connectedness, Input–output linkage, C30, E16, E44, G11, G12

## Abstract

This paper examines the shock spillovers between US sectors and their dependence on the intersectoral business linkages. Our forecast error variance decompositions reveal significant shock transmissions among trading sectors, especially in turbulent periods such as the financial crisis and the COVID-19 pandemic. The dymamics of shock spillovers reflect the impacts of the pandemic on economic sectors. Shock spillovers are shown to be influenced by the strength of the intersectoral trading relationships. Shocks to a sector’s important supplier have a strong impact on the forecast error variance of the sector’s stock return. The total directional spillovers from/ to a sector are linked with the number of close commercial linkages between that sector and other sectors.

**Supplementary Information:**

The online version contains supplementary material available at 10.1007/s10479-022-04979-8.

## Introduction

The Global Financial Crisis (GFC) and the COVID-19 pandemic have drawn renewed attention to shock transmissions in the economy across both production and financial networks. The economics literature has shown the possibility of “cascade effects”, whereby sectoral shocks propagate to direct trading partners and also to the aggregate economy. Acemoglu et al. (2012) show that input–output connections between sectors act as a potential propagation mechanism of idiosyncratic shocks throughout the entire economy.^1^ Carvalho (2014) demonstrates the importance of the structure of a production network: in an economy characterized by a small number of production hubs supplying inputs to many different firms or sectors, microeconomic shocks tend to result in higher aggregate volatility. Moreover, Atalay (2017) estimates that sectoral shocks account for nearly two thirds of the volatility of aggregate output.^2^

In the same vein, the finance literature has provided ample evidence of the connectedness between financial markets. The co-movements of stock returns and volatility between international stock markets have been documented by Hamao et al. (1990), King and Wadhwani (1990), Diebold and Yilmaz (2009), Zhu et al. (2018), Jawadi et al. (2019), inter alia. Other studies examine shock spillovers among related industries within specific sectors such as energy (Alli et al., 1994; Ewing et al., 2002) and financials (Elyasiani et al., 2007). Wang (2010) investigates the dynamics and causality among 30 US industries to identify leading-lagging industries. Neither of these studies, however, consider the underlying economic relationships as factors potentially driving the uncovered spillovers.

Our paper bridges these two lines of research. We quantify the shock spillovers of stock returns among *all* the economic sectors in the US economy. In this respect, we generalize—to the best of our knowledge, for the first time—the specific, intra-sectoral perspective so far offered by the financial studies. We then investigate how the intensity of the said financial spillovers depends on the strength of the intersectoral trade relationships, as captured by the set of input–output connections among all the sectors.

The first part of our analysis documents the existence and the intensity of financial shock spillovers among all the US non-governmental sectors. We employ a technique proposed by Diebold and Yilmaz (2012, 2014) to analyse the daily stock returns of 14 US economic sectors. The approach, which decomposes the forecast error variance under a generalized vector autoregressive (VAR) framework, allows us to evaluate the degree to which shocks to stock returns are transmitted: (1) between pairs of sectors; (2) from a sector to the rest of the market; and (3) from the rest of the market to a sector. The technique can also be used to compute the extent of total spillovers, which measures the overall degree of connectedness among all the sectors in the economy.^3^ We augment the Diebold and Yilmaz (2012, 2014) approach with exogeneous variables and estimate a VARX system which accounts for macroeconomic factors, stripping the spillover measures from the common macroeconomic effects which are likely to affect all the sectors in the economy.^4^ To gauge how shock spillovers propagate across sectors and evolve over time, we conduct both a static, full sample, and a dynamic, rolling-sample, analysis.

The second important contribution of this paper is to investigate the relationship between the intensity of shock spillovers, uncovered in the first stage, and the strength of the trade links between the sectors. We are motivated by recent empirical evidence which suggests that economic links explain contemporaneous correlations between firms’ stock returns and at the same time help predict future stock returns (Cohen & Frazzini, 2008; Menzly & Ozbas, 2010). To quantify the strength of the economic link between sectors, we follow Becker and Thomas (2011) and Ahern and Harford (2014) by using information from the Input–Output (IO) tables published by the US Bureau of Economic Analysis (BEA).^5^

As a preview of our variance decomposition results, we observe significant shock transmission between US sectors. The total shock spillover among the 14 sectors in our sample is 85.8%. Shocks to a sector are found to account for up to 10.16% of the forecast error variances of its trading partners’ stock returns. Compared to the average sector’s own contribution of 14.2%, this cross variance share is considerably large. All shock spillover measures remain at high levels when the spillovers are measured both statically, using the full sample period, and dynamically, over the 200-day rolling windows.

The second part of our analysis confirms that business linkages have significant effects on shock transmissions between sectors: the closer the trading relationship in a sector pair, the stronger the degree of spillovers. Shocks to a sector’s important supplier account for a large fraction of the forecast error variance of the sector’s return. Additionally, we show that the total directional spillovers *from* and *to* a sector correlate with the number of close linkages between that sector and the others: a sector which is a main supplier/customer to many other sectors is likely to be a net shock transmitter in the network.

An interesting additional result is that the intersectoral shock transmission and its dependence on the trade links are affected by the market conditions. In particular, our analysis conducted on sub-samples representing different market conditions shows stronger shock transmissions between sectors during periods of stress such as the GFC and the COVID-19 pandemic.

We check for the robustness of our results by subjecting them to a series of sensitivity tests. Firstly, we find similar evidence of significant shock spillovers among sectors when we conduct our analysis on a sample including only non-financial sectors. Secondly, we show that the magnitude of the shock spillovers is insensitive to the order of the VARX model and the choice of the forecast horizon. Finally, the relationship between shock spillovers and the strength of the business linkages between sectors is robust to an alternative construction of the business linkage variables.

Our results provide important implications for investors and portfolio managers with exposure to specific sectors. Sector funds, such as agriculture or energy funds, are attractive to investors who want to focus their knowledge on a manageable set of companies with similar business operations. Several studies have reported the outperformance of sector fund strategies. Chen and Hackbarth (2020) use the data of active equity sector funds and show that a naïve equal-weighted portfolio provides reliable alpha and the outperformance is resilient in market downturns, with infrequent need of rebalancing. O’Neal (2000) finds that sector mutual funds have profitable trading strategies based on sector momentum effect. However, these sector-specific investment strategies entail greater total and systematic risk than index. Thus, for better risk management, our study suggests that practitioners should pay close attention to sectors with close business linkages because returns and volatility of their investments could be affected indirectly by shocks to the related sectors.

Understanding shock transmissions across sectors also has important policy implications. A policy change targeting a particular sector could potentially have considerable impacts on other sectors given their high degree of connectedness. Moreover, since our results show that sectors in the US economy are closely connected, large systemic idiosyncratic shocks hitting several sectors simultaneously could potentially have a devastating negative impact on the financial system especially during turbulent periods.

Our study also contributes to the recent literature strand on the financial impacts of COVID-19 (see Corbet et al., 2020; Iqbal et al., 2021, among others). Akhtaruzzaman et al. (2021) report a significant increase in volatility and shock transmissions between stock markets during the COVID-19 outbreak. Sharif et al. (2020) employ a wavelet coherence approach and show that the pandemic is expected to have a long-term negative impact on the US economic uncertainty. A similar approach is used by Choi (2020) to investigate the effect of the COVID-19 on US sectors. These studies reveal that the economic policy uncertainty induced by COVID-19 has greater influence on all sectors volatility, compared to the effect of GFC. However, the impact of COVID-19 on the shock spillovers between sectors has not been examined. Thus, our work provides insights into sectoral shock transmissions during the pandemic, which are valuable for policy makers, regulators and practitioners.

Our paper is structured in three main parts. Part I investigates the degree of connectedness among the US economic sectors. Section 2 describes the generalized variance decomposition methodology, Sect. 3 discusses the sector stock return data and summary statistics, and Sect. 5 reports the empirical results of the shock transmissions between sectors. Part II extends the analysis to the relation between inter-sectoral shock spillovers and the strength of their business linkages (Sect. 5). Part III reports additional results. Section 6 examines the shock transmissions and the impact of business linkages in different market conditions. Section 7 collects our robustness checks. Finally, Sect. 8 concludes.

**Part I: Shock Spillovers among the US Sectors**

## Methodology

To investigate the transmission of shocks, we follow Diebold and Yilmaz (2012, 2014) to use a generalized vector autoregression (VAR) system for $N$ sectors in the economy, and measure the forecast error variance decompositions across sectors. To account for the common impact of macroeconomic factors on sector returns, we extend VAR to include exogenous variables (see also Alter & Beyer, 2014; Claeys & Vašíček, 2014). Specifically, consider a covariance stationary VARX(*p*) system of $N$ economic sectors*:*

1 $$ \user2{y}_{t}  = \sum\limits_{{k = 1}}^{p} {{\mathbf{\Phi }}_{k} } \user2{y}_{{t - k}}  + \user2{b}^{\prime } \user2{x}_{t}  + \user2{\varepsilon }_{t}, $$

where ${{\varvec{y}}}_{t}=\left({y}_{1t},{y}_{2t},\dots ,{y}_{Nt}\right){^{\prime}}$ is an $N\times 1$ vector of sector excess returns at time $t$, ${{\varvec{\varepsilon}}}_{t}\sim \left(0,{\varvec{\Sigma}}\right)$ is a vector of the error terms at time $t$, which are independently and identically distributed with zero mean and variance–covariance matrix ${\varvec{\Sigma}}$**,** and ${{\varvec{x}}}_{t}$ is a vector of exogenous macroeconomic variables at time *t*. The corresponding moving average representation of the VAR(*p*) system in Eq. (1) is

2 $${{\varvec{y}}}_{t}=\sum_{k=0}^{\infty }{{\varvec{A}}}_{k}{{\varvec{\varepsilon}}}_{t-k},$$

where the $N\times N$ coefficient matrix ${A}_{k}$ is calculated recursively:

${{\varvec{A}}}_{k}={{\varvec{\Phi}}}_{1}{{\varvec{A}}}_{k-1}+{{\varvec{\Phi}}}_{2}{{\varvec{A}}}_{k-2}+\cdots +{{\varvec{\Phi}}}_{p}{{\varvec{A}}}_{k-p}$ for $k>0$, ${{\varvec{A}}}_{k}$ is an identity matrix for $k=0$, and ${{\varvec{A}}}_{k}=0$ for $k<0$.

The moving average coefficients, known as variance decompositions, reveal the dynamics of the system. Variance decompositions show the fraction of the $H$-step-ahead error variance in forecasting ${y}_{i}$ accounted for by the shocks to ${y}_{j}$, $\forall j\ne i$, for each $i$ in the system. Since VAR innovations are generally contemporaneously correlated, the variance decompositions traditionally rely on identification schemes such as those based on Cholesky factorization to achieve orthogonality (Sims, 1980). Using the traditional approach, however, renders variance decompositions dependent on the ordering of the variables in the VAR system. Diebold and Yilmaz (2012, 2014) overcome the ordering-dependence problem by employing the generalized VAR framework proposed by Koop et al. (1996) and Pesaran and Shin (1998). Instead of orthogonalizing the shocks, the framework uses the historically observed distribution of the errors to account for the correlated shocks.

The coefficient matrix ${{\varvec{A}}}_{k}$ contains a large amount of useful information which can be extracted as follows. Firstly, using the elements in ${{\varvec{A}}}_{k}$, we calculate the cross variance shares, or *pairwise directional spillovers*, which are defined as the proportion of the $H$-step-ahead forecast error variance of sector $i$’s return attributable to the shocks to sector $j$ ($i,j=\mathrm{1,2},\dots ,N$; $i\ne j$). When $i=j$, the measure is termed the *own variance shares* which are defined as the proportion of the forecast error variance of sector $i$’s return accounted for by its own shocks. According to the generalized VAR framework, the $H$-step-ahead forecast error variance decomposition is obtained as

3 $${\theta }_{ij}^{H}=\frac{{\sigma }_{jj}^{-1}\sum_{h=0}^{H-1}{\left({{\varvec{e}}}_{i}^{^{\prime}}{{\varvec{A}}}_{h}{\varvec{\Sigma}}{{\varvec{e}}}_{j}\right)}^{2}}{\sum_{h=0}^{H-1}\left({{\varvec{e}}}_{i}^{^{\prime}}{{\varvec{A}}}_{h}{\varvec{\Sigma}}{{{\varvec{A}}}_{h}^{^{\prime}}{\varvec{e}}}_{i}\right)}$$

where ${\varvec{\Sigma}}$ is the variance–covariance matrix of the shock vector in the VAR system, ${\sigma }_{jj}$ is the ${j}^{th}$ element of the diagonal of ${\varvec{\Sigma}}$ representing the standard deviation of the shock for the ${j}^{th}$ sector, ${{\varvec{e}}}_{i}$ is the selection vector with one as the ${i}^{th}$ element and zeros elsewhere, and ${{\varvec{A}}}_{h}$ is the coefficient matrix in the infinite moving average representation in Eq. (2), with $h=0, 1, 2\dots , H-1$. Since shocks to the sectors in the system can be contemporaneously correlated, the sum of all the contributions to a sector’s variance (i.e., the sum of all the elements in each row of the variance decomposition table) can differ from one (i.e., ${\sum }_{j=1}^{N}{\theta }_{ij}^{H}\ne 1)$. For this reason, Diebold and Yilmaz (2012, 2014) normalize entries in the variance decomposition matrix by dividing the quantity in Eq. (3) by the row sum:

4 $${S}_{i\leftarrow j}^{H}={\widetilde{\theta }}_{ij}^{H}=\frac{{\theta }_{ij}^{H}}{\sum_{j=1}^{N}{\theta }_{ij}^{H}}$$

Note that ${\sum }_{j=1}^{N}{\widetilde{\theta }}_{ij}^{H}=1$ and ${\sum }_{i,j=1}^{N}{\widetilde{\theta }}_{ij}^{H}=N$ by construction. In our investigation, we refer to ${S}_{i\leftarrow j}^{H}$ as the pairwise spillover from sector $j$ to sector $i$ after $H$ periods. For a system of $N$ sectors, there are ${N}^{2}-N$ pairwise spillover measures in total.

Secondly, in addition to the measure of pairwise spillovers, we also calculate the *total directional spillover from others* to sector $i$ as5and the *total directional spillover* to others from sector $i$ as6

The *net directional spillovers* from sector $i$ to all other sectors can then be easily calculated from Eqs. (5) and (6) using7

Finally, the *total spillover* between all sectors in the system can be obtain as

8 $${S}^{H}=\frac{1}{N}\sum_{\begin{array}{c}i,j=1\\ i\ne j\end{array}}^{N}{\widetilde{\theta }}_{ij}^{H}$$

which is the equal-weighted average of the sum of all the entries, excluding the own variance shares, in the variance decomposition matrix.

## Data and summary statistics

To calculate sector excess returns, we use daily stock returns for all the stocks listed on four US major stock markets—NYSE, Nasdaq, Arca and Amex with share code 10 and 11—obtained from the CRSP database. We sort all stocks into 14 sectors using the NAICS codes. Our dataset spans the period starting from 3rd January 2005 to 31st December 2020, giving us a total of 4028 daily observations for each of the sectors.^6^ Daily sector returns are calculated as the sum of the value-weighted returns of all the stocks in the sector, where the weights are the beginning-of-the-day market capitalization of the stocks. The sector excess return is computed as the difference between the sector return and the yield on the 3-month US Treasury bills, obtained from the Federal Reserve Bank of St. Louis (FRED) database.

To account for macroeconomic factors, we use the short-term risk-free rate, the term risk premium (the difference between the 10-year Treasury bond yield and the 3-month T-Bill yield), the credit risk premium (the difference between Moody’s Baa corporate bond yield and Moody’s Aaa corporate bond yield) and the foreign exchange rate index. We obtain the data on the 3-month T-bill yield, 10-year Treasury yield, Moody’s Baa and Aaa corporate bond yield, and the trade weighted USD indices against a broad group of major US trading partners from the Federal Reserve Bank of St. Louis (FRED) database. The Augmented Dickey-Fuller (ADF) test suggests the use of the first-differences of the yields and the foreign exchange rate index, and confirms the stationarity of all the 14 sector excess return series.

**Fig. 1:**
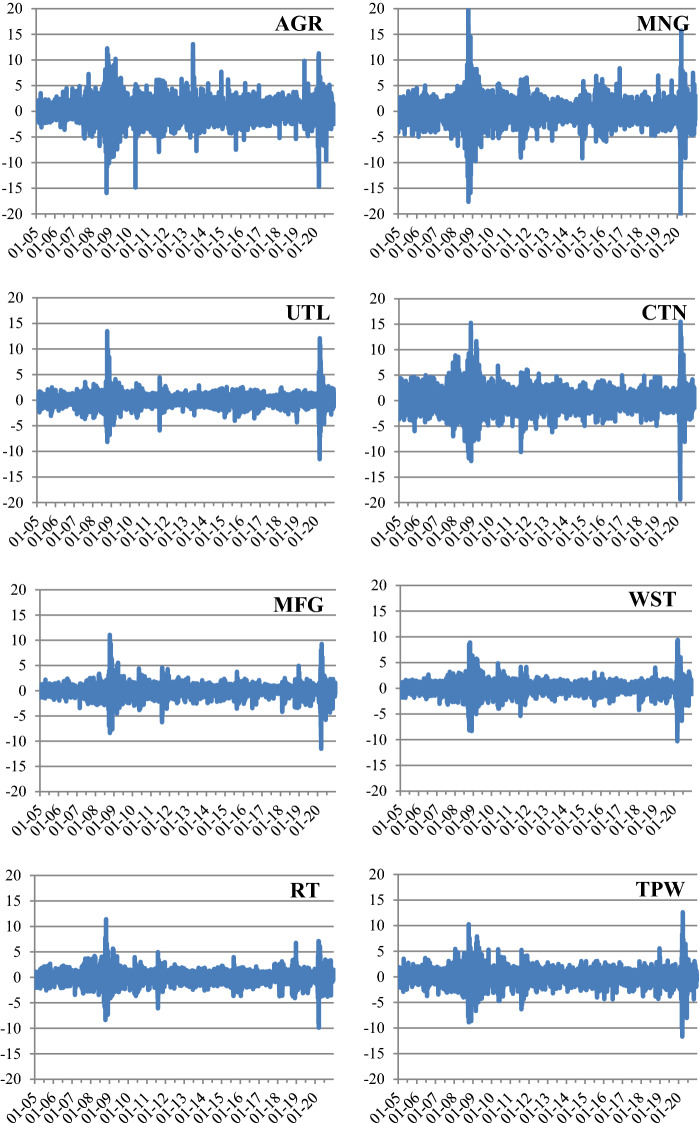

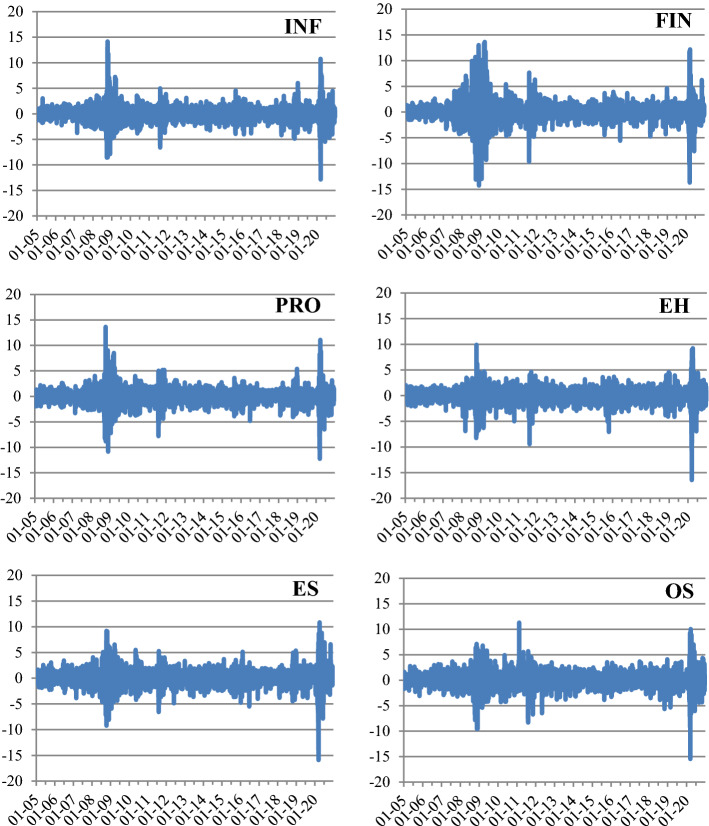
Sector stock returns. This figure illustrates the time series of 14 sector excess returns over the period 3 January 2005–31 December 2020

Table 1 presents the descriptive statistics of our data. The mean daily excess returns range from 0.033% (AGR and MNG) to 0.061% (INF). Among the 14 sectors, Mining (MNG) has the largest standard deviations. All the graphs of the sector excess returns in Fig. 1 show a common pattern of a significant increase in volatility following the GFC, especially after the collapse of Lehman Brothers in September 2008, and during the pandemic in 2020.

**Table 1:** Summary statistics of the sector returns

No	Sector	Abbrevia-tion	Mean (%)	Median (%)	Max (%)	Min (%)	Std.Dev (%)	Skewness	Kurtosis
1	Agriculture, forestry, fishing, and hunting	AGR	0.033	0.046	13.101	− 15.939	2.015	− 0.142	6.409
2	Mining	MNG	0.033	0.071	21.383	− 23.463	2.203	− 0.376	11.253
3	Utilities	UTL	0.040	0.094	13.487	− 11.543	1.194	0.283	18.578
4	Construction	CTN	0.038	0.042	15.509	− 19.387	2.089	− 0.115	7.086
5	Manufacturing	MFG	0.049	0.094	11.088	− 11.519	1.193	− 0.317	11.992
6	Wholesale trade	WST	0.047	0.073	9.401	− 10.313	1.198	− 0.260	9.737
7	Retail trade	RT	0.055	0.089	11.421	− 9.897	1.213	− 0.064	8.088
8	Transportation and warehousing	TPW	0.051	0.098	12.638	− 11.686	1.483	− 0.291	7.588
9	Information	INF	0.061	0.096	14.164	− 12.861	1.322	0.014	13.045
10	Finance, insurance, real estate, rental, and leasing	FIN	0.034	0.067	13.635	− 14.280	1.777	0.044	13.039
11	Professional and business services	PRO	0.051	0.100	13.641	− 12.255	1.406	− 0.203	11.122
12	Educational services, health care, and social assistance	EH	0.042	0.092	9.903	− 16.453	1.346	− 0.795	12.565
13	Arts, entertainment, recreation, accommodation, and food services	ES	0.055	0.090	10.855	− 15.903	1.527	− 0.501	9.327
14	Other services, except government	OS	0.041	0.077	11.307	− 15.460	1.512	− 0.474	9.389

This table provides descriptive statistics of the excess return time series of 14 sectors over the period 3 January 2005–31 December 2020, with a total of 4028 daily observations. Daily sector returns are calculated as the sum of value-weighted returns of all the stocks in the sector, where the weights are the beginning-of-the-day market capitalization of the stocks. The sector excess return is computed as the difference between the sector return and the risk-free rate, proxied by the yield on the 3-month US Treasury bills

## Shock spillovers among the US sectors

This section reports the results of the investigation of shock spillovers between sectors using the generalized variance decomposition method. We conduct both a static analysis for the full sample period and a rolling sample (dynamic) analysis of conditional connectedness.

### Static analysis: full-sample spillovers

We first estimate the VARX model for the daily excess returns of a system of 14 sectors using the full sample from 2005 to 2020. Following Diebold and Yilmaz (2012, 2014), the forecast error variance across sectors is decomposed into parts attributable to the various variables in the system. The variance decomposition matrix, also known as the *connectedness table*, is shown in Table 2. The table reports the magnitude of the pairwise, directional and total spillovers, calculated from the first-order VARX at the 10-day-ahead forecast horizon.^7^

**Table 2:** Full-sample Shock spillover

	AGR	MNG	UTL	CTN	MFG	WST	RT	TPW	INF	FIN	PRO	EH	ES	OS	FROM others
AGR	19.69	4.39	4.19	6.43	7.44	7.19	5.96	6.85	6.26	7.04	7.77	5.03	6.11	5.65	80.31
MNG	4.04	18.44	5.76	6.62	9.42	7.46	4.38	7.67	6.28	6.10	7.81	4.96	5.97	5.08	81.56
UTL	3.77	5.54	18.01	5.13	9.44	8.80	5.96	6.55	7.26	5.99	7.89	5.46	5.05	5.16	81.99
CTN	4.63	5.10	4.07	14.20	8.01	7.70	6.73	7.94	6.45	7.88	8.47	5.54	7.16	6.12	85.80
MFG	4.03	5.58	5.73	6.04	10.94	8.84	7.47	7.61	8.91	6.92	9.29	6.07	6.81	5.78	89.06
WST	4.22	4.82	5.84	6.28	9.59	11.65	7.29	7.88	7.72	7.10	8.81	5.91	6.67	6.22	88.35
RT	4.01	3.35	4.55	6.34	9.36	8.42	13.30	7.31	9.22	6.69	8.78	5.68	7.26	5.72	86.70
TPW	4.29	5.24	4.66	6.95	8.88	8.50	6.81	12.44	7.21	7.40	8.58	5.57	7.67	5.80	87.56
INF	3.82	4.33	5.00	5.50	10.16	8.10	8.35	7.05	12.34	6.85	9.88	5.76	7.18	5.69	87.66
FIN	4.63	4.40	4.41	7.22	8.39	8.01	6.48	7.72	7.31	13.21	9.72	5.50	6.95	6.05	86.79
PRO	4.25	4.66	4.82	6.46	9.41	8.26	7.11	7.48	8.79	8.11	11.04	6.17	7.14	6.29	88.96
EH	3.82	4.11	4.68	5.95	8.64	7.79	6.46	6.84	7.17	6.45	8.66	15.35	7.12	6.96	84.65
ES	4.08	4.37	3.85	6.65	8.46	7.70	7.20	8.18	7.81	7.13	8.73	6.15	13.17	6.50	86.83
OS	4.22	4.13	4.33	6.37	8.05	8.00	6.36	6.92	6.99	6.93	8.64	6.78	7.32	14.95	85.05
TO others	53.80	60.02	61.88	81.94	115.25	104.78	86.57	96.00	97.38	90.59	113.04	74.58	88.41	77.03	**85.80**
NET	− 26.51	− 21.54	− 20.11	− 3.86	26.20	16.42	− 0.13	8.44	9.72	3.80	24.08	− 10.07	1.58	− 8.02	

This table reports Shock spillovers between 14 sectors using the full sample from 3 January 2005 to 31 December 2020. The $ij$*-*th element of the upper-left $14\times 14$ submatrix reports the $ij$*-*th pairwise spillover in percentage, i.e., the fraction of 10-day-ahead forecast error variance of sector $i$ accounted for by shocks to sector $j$*.* The rightmost column, FROM others, shows the total directional spillovers from all other sectors to a sector *i* (sum of off-diagonal entries in row $i$). The bottom row, TO others, shows the total directional spillovers from sector $j$ to all other sectors (sum of off-diagonal entries in column $j$). The bottommost row (NET) shows the difference between the total directional spillovers TO and FROM other sectors to a specific sector. The bottom-right element (in boldface) gives the total spillovers between sectors

The pairwise spillovers between sectors are given in the off-diagonal elements of the $14\times 14$ matrix shown in Table 2, while elements along the diagonal axis show the contribution of a sector to its own forecast variance. The own connectedness (diagonal elements) tends to account for the largest share of each sector’s forecast error variance, ranging from 10.94% Manufacturing (MFG) to 19.69% Agriculture, forestry, fishing, and hunting (AGR). The average cross variance share (off-diagonal elements) is 6.60%, showing that a sizable proportion of the forecast error variance of sector $i$ comes from shocks to sector $j$. The highest pairwise spillover, is 10.16%, from Manufacturing (MFG) to Information (INF). In contrast, the cross variance share of 3.35%, contributed by Mining (MNG) to Retail trade (RT), is the smallest.^8^

The total directional spillovers (from others and to others) tend to be large pointing to strong connections among sectors. The total directional spillovers to a sector from all the other sectors (the row sum excluding the diagonal element) are reported in the rightmost column labelled “FROM others”. The total directional spillovers from a sector to all the others (the column sum) are reported along the bottom row labelled “TO others”.^9^ According to the results presented in Table 2, the total directional spillover coming from other sectors varies between 80.31 and 89.06%, while the total directional spillover originating from a sector to the others ranges from 53.80 to 115.25%. Agriculture, forestry, fishing, and hunting (AGR) is found to be both the smallest shock transmitter and receiver. At the same time, Manufacturing (MFG), Professional and Business Services (PRO), and Wholesale Trade (WST) are the top three shock transmitters and receivers. These three sectors account for both the largest total directional spillovers to others (over 100%) and received from others (nearly 90%).

**Fig. 2:**
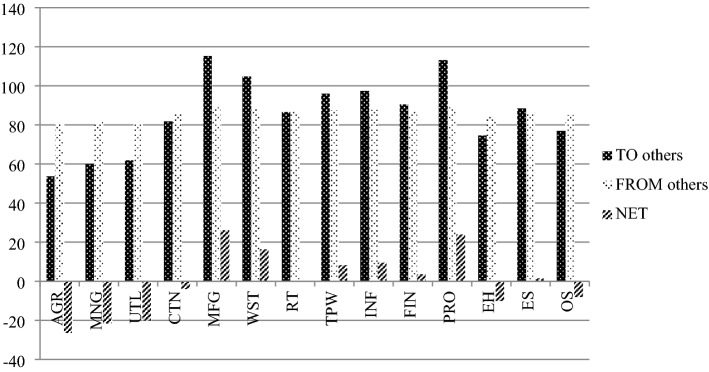
Directional spillovers in 2005–2020. This figure illustrates the total directional spillover from a sector TO others, the total directional spillover FROM others to a sector, and the net directional spillover of a sector. The measures are obtained from the generalized variance decomposition approach over the period 3 January 2005–31 December 2020

For each of the 14 sectors, we calculate the net total directional spillovers (NET) as the difference between the total spillover from a sector “TO others” and the total spillover it receives “FROM others”. The NET values are reported along the last row of Table 2. It appears that Manufacturing (MFG), Professional and business services (PRO), and Wholesale Trade (WST) are the largest transmitters, receivers, and have the largest positive net total directional spillovers (26.20%, 24.08% and 16.42%, respectively). At the other extreme, Agriculture, forestry, fishing, and hunting (AGR), Mining (MNG), and Utilities (UTL) stand out as the three major net shock receivers in the network, as shown by their corresponding large negative net total directional spillovers (− 26.51%, − 21.54%, and − 20.11%, respectively). Figure 2 provides a graphical illustration of the total directional spillovers (TO, FROM, NET) by sector.

Finally, the total shock spillover among all the sectors is estimated to be 85.8%. Our estimate for the total connectedness among the US sectors is substantial but compares well with the 78% total connectedness index for 13 major US financial institutions in Diebold and Yilmaz (2014), and the 82% index of connectedness for nine equity markets in Diebold and Yilmaz (2015). Yarovaya et al. (2016) report a total shock transmission of 71% between 21 stock markets.^10^ Taken together, the results reported in this section point to significant connectedness among the 14 US economic sectors.

### Dynamic analysis: rolling-sample spillovers

The analysis in Sect. 4.1 employs information over the whole 2005–2020 period to calculate unconditional shock spillover measures among the 14 US sectors. While our previous findings reveal strong connectedness among the US sectors, they do not uncover the dynamics of the spillovers, i.e., how the connectedness evolves over time. During the 16-year period under examination, the US economy went through different market conditions and experienced a number of major shocks. The impacts of both the GFC and COVID-19 on the US stock market prompted the Federal Reserve to cut its policy rate to near zero and embark on large scale asset purchases to prop up the US economy. The COVID-19 pandemic also has profound negative impacts on the world economy as lockdown restrictions caused consumer demand on non-essential items to evaporate. It is possible that the nature of spillovers among the 14 sectors differed during these turbulent periods. We examine this conjecture by estimating the spillover measures over 200-day rolling-sample windows.^11^

**Fig. 3:**
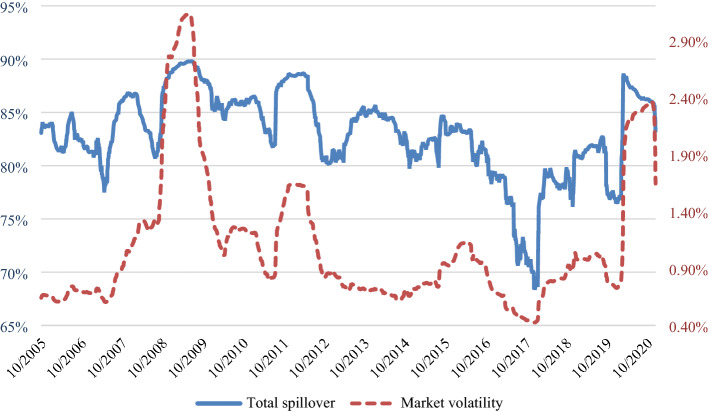
Total spillover—rolling windows. This figure plots the total spillover between 14 sectors and market volatility over 200-day rolling sample windows. The horizon axis shows the ending dates of the rolling samples

Figure 3 plots the time series of the total spillover measure over the rolling windows, alongside the volatility of the US stock market, measured as the standard deviation of the daily returns of the CRSP index over the corresponding 200-day windows. At first glance, the plot suggests that the total spillover between sectors remains considerably large at around 70–90% over the entire 2005–2020 period. It also reveals distinguished patterns across different market conditions. The first cycle spans the pre-crisis period 2005–2006 where the economic climate was relatively calm and inflation was low and stable, with the estimated total spillover of about 80% to 85%. The second episode reflects the large fluctuations during the financial crisis where the total spillover between sectors surges to nearly 90% at the end of 2008—the beginning of 2009 and market volatility peaks. Despite some ups and downs, the total spillover persists at this high level after the crisis and peaks once more in 2012, coinciding with the implementation of the third round of quantitative easing (QE3). The third cycle spans the second half of 2012–2016 when the total spillover reverses to its pre-crisis level of around 80–85%. The total spillover experiences a decline to just below 70% along with the lowest level of market volatility in 2017 and remains rather stably at around 70–80% during the calm market in 2018–2019. The last cycle observes a jump in the total spillover to nearly 90%, together with a high level of market volatility following the COVID-19 outbreak at the beginning of 2020. Both total spillover and market volatility decrease at the end of 2020 along with the vaccination rollout. Overall, we observe substantial total spillovers across the rolling windows. It is clear that the total spillovers among the 14 US sectors exhibit a time-varying nature, where shock spillovers tend to increase in turbulent market conditions.

**Fig. 4:**
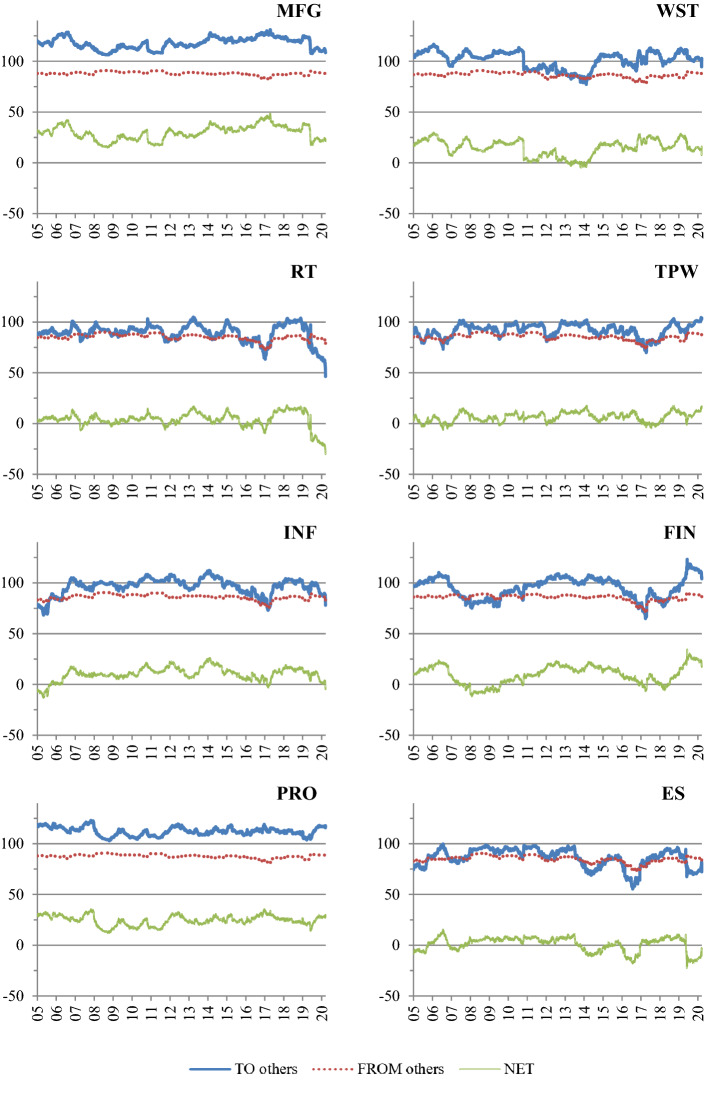

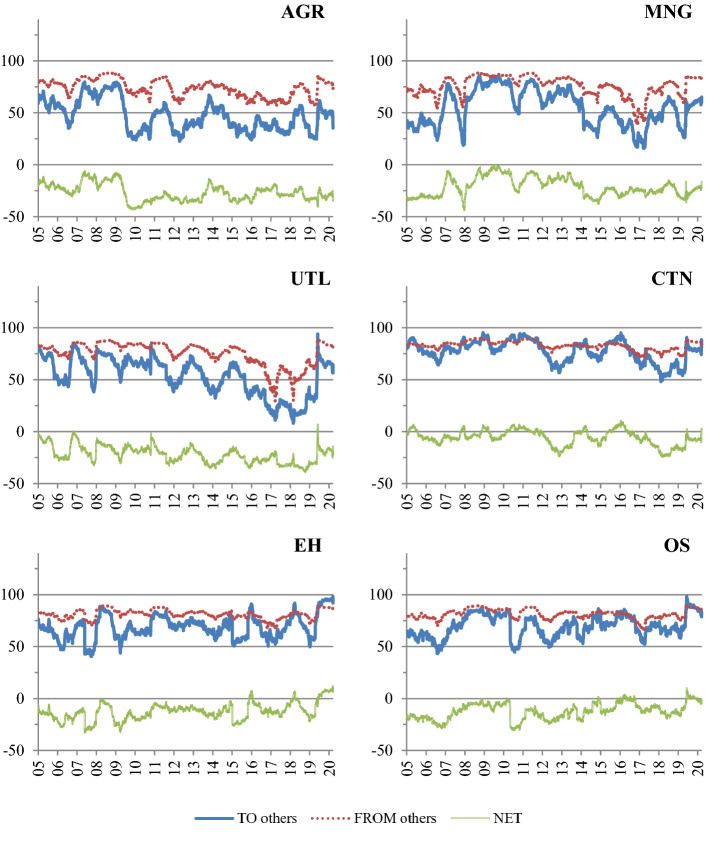
Directional spillover—rolling windows. This figure plots the total directional spillovers from a sector to others, the total directional spillover from others to a sector and the net directional spillovers of 14 US sectors over 200-day rolling sample windows

For each of the 200-day rolling windows, we calculate the total directional spillovers from a sector “TO others”, “FROM others” to a sector, and the “NET” directional spillovers, which are computed as the difference between the first two spillover measures. Figure 4 plots the time series of these measures by sector.^12^ For the purpose of presenting our results, we separate the 14 sectors into two groups. Figure 4.1 illustrates the results for the net shock transmitters, i.e., the sectors with (generally) positive NET total directional spillovers. Figure 4.2 refers to the net shock receivers, i.e., the sectors with (generally) negative NET total directional spillovers. While the “FROM” plots appear to be relatively smooth, the total spillovers from a sector “TO others” exhibit noticeably larger variation over time. This seems to suggest that shocks transmitted to a sector from other sectors wash out in aggregate as argued by Lucas (1977). Different patterns emerge when we compare the plots of the shock transmitters to those of the shock receivers. We observe that the “FROM” plots in Fig. 4.1 are smoother than the corresponding plots in Fig. 4.2. This implies that returns for sectors that are net shock receivers (e.g., Agriculture, forestry, fishing and hunting—AGR and Mining—MNG) are likely to be more susceptible to external shocks compared to returns for sectors that are net shock transmitters, such as Manufacturing (MFG).

The dynamics of the total directional spillovers also reflect how the COVID-19 pandemic has changed the way the world operates. Retail Trade (RT), which is usually a shock transmitter, experiences the largest decrease in shock transmission to other sectors in 2020, and becomes a shock receiver with the NET total directional spillover of almost -30%. In contrast, we observe an increase in the total directional spillovers from Transportation and warehousing (TPW) and Finance, insurance, real estate, rental and leasing (FIN) “TO others”. The Educational services, health care, and social assistance (EH) sector also switches from a shock receiver to a shock transmitter, with a significant increase in the total directional spillovers “TO others”. More detailed discussion of the pairwise spillovers between sectors during COVID-19 is provided in our sub-sample analysis in Sect. 6.

**Part II: Business linkages and shock spillovers**

## The strength of the business linkages and shock spillovers between sectors

All the results reported in Part I point to significant shock spillovers among the 14 US sectors. In Part II, we extend the error variance decomposition analysis by examining if the trade flows between pairs of sectors can explain the degree of return spillovers. Our hypothesis is that the financial connectedness observed in our data is driven by the commercial linkages between sectors, i.e., larger trade flows between sectors are associated with larger shock spillovers.

To formally test our hypothesis, we first construct variables which measure the strength of the trade linkages between each pair of sectors. Such measures based on data from the Input–Output (IO) accounts have been used in a number of finance studies. Menzly and Ozbas (2010), for instance, investigate the return spillovers between supplier and customer industries. They use the trade flows between industries, obtained from the IO tables, as weights to construct two separate portfolios of the representative supplier and the representative customer for each industry. Their results suggest that returns on an industry are affected by the lagged returns of its representative supplier and customer industries. Aobdia et al. (2014) also find significant return interdependence between an industry and its “related” industry, which is constructed as a portfolio of all its trading partner (both supplier and customer) industries. Ahern (2013) finds that return spillovers between industries are affected by the closeness of the relationships. Shocks to an industry return have immediate effects on its closely-related industries and delayed effects on its distant-connected industries. He also demonstrates that a central industry has greater exposure to market risk than a distant one. As a result, its stock returns comove more closely with the market returns. Ahern and Harford (2014) utilize the IO data to create a network of suppliers and customers and gauge the strength of the links between industries. They find that merger activities spill over the supplier-customer network in wave-like patterns.

Details of the construction of these variables and some descriptive statistics are discussed in Sect. 5.1 below. In what follows, we perform a series of cross-sectional regressions of the spillover measures, calculated from the full sample, on the measures of business linkages. Section 5.2 presents the results regarding the pairwise spillovers, while Sect. 5.3 analyzes how the importance and closeness of a sector to the others affect the total directional spillovers.

### Business-linkage measures

We use the information from the IO accounts, provided by the Bureau of Economic Analysis (BEA), to measure the strength of economic linkages between pairs of sectors. These accounts report the value of commodities (goods and services) produced and traded among all the sectors in the US economy. BEA use information from the Economic Census to provide benchmark IO tables every five years (i.e., years ending 2 and 7) and estimated tables for the other years. In this paper, we use the IO accounts aggregated at the sector level, which consist of 15 sectors and 17 commodities. Our analysis includes the 14 non-governmental sectors which have been used in the variance decomposition analysis discussed above.

#### Measure construction

Our measures of economic linkages between pairs of sectors are calculated based on the information from the two main IO tables: the *Make* and the *Use* tables (for snapshots of these tables see Appendix). The *Make* table reports the value of each commodity produced by sectors. In this table, each row presents a sector while commodities are shown across columns. It is noteworthy that, although a commodity is mainly produced by a sector, the same commodity can also be supplied by other sectors. Equivalently, while a sector predominantly produces one commodity, it may also supply other commodities to other sectors. Thus, the total output of a sector, denoted by ${OUTPUT}_{i}$, is calculated as the sum of all the entries along the corresponding row, while the total output of a commodity produced by all the sectors is computed as the sum of all the entries in the corresponding column.

The *Use* table reports the value of each commodity purchased as an input for the production of other sectors (or consumed by final users). Elements along each row in this table show the commodity output while elements in each column present the sector input. Therefore, the total commodity output is computed as the sum of all the entries along the corresponding row while the total input value for a sector, denoted by ${INPUT}_{j}$, is calculated as the sum of all the commodity entries in the respective column. The sum of the total sector input and the total value added is the total sector output (${OUTPUT}_{j}$), presented in the last row of the *Use* table.

Following Ahern and Harford (2014) and Becker and Thomas (2011), we use the trading values of the commodities reported in the *Make* and the *Use* tables to construct the *CUST* and the *SUPP* matrices. These matrices quantify the importance of sectors as customers and suppliers to one another, respectively. Using the information in the *Make* table, we first calculate the subordinate *SHARE* matrix which shows the share of each sector’s commodity output in the total supply of each commodity. Specifically, the element in row $i$, column $c$ in the *SHARE* matrix, denoted ${SHARE}_{ic}$, is computed as:

9 $${SHARE}_{ic}=\frac{{Make}_{ic}}{{Total\,Supply}_{c}}$$

where $i$ and $c$ represent sector and commodity, respectively. The numerator ${Make}_{ic}$ shows the value of commodity $c$ produced by sector $i$ (element in row $i$, column $c$ of the *Make* table) while the denominator ${Total Supply}_{c}$ is the total supply of commodity $c$, which is the total output of commodity $c$ produced by all sectors in the economy (i.e., the sum of all entries in the commodity $c$ column in the *Make* table) plus other components such as imports or changes in inventories.

Using elements from the *SHARE* matrix along with the information from the *Use* table, we construct the *REVSHARE* matrix, which contains the value of commodities transacted between sectors. The element in row $i$, column $j$ of the matrix, denoted by ${REVSHARE}_{ij}$, shows the total value of all the commodities which sector $j$ purchases from sector $i$ and is given by:

10 $${REVSHARE}_{ij}=\sum_{c=1}^{C}\left({SHARE}_{ic}\times {Use}_{cj}\right)$$

where ${SHARE}_{ic}$, defined previously in Eq. (9), is the proportion of commodity $c$ produced by sector $i$ (i.e., element in row $i$, column $c$ of the *SHARE* matrix) and ${Use}_{cj}$ (i.e., the element in row $c$, column $j$ of the *Use* table) presents the value of commodity $c$ purchased by sector $j$.^13^ The commodity flows between sectors, obtained from the *REVSHARE* matrix, are the key ingredients in the construction of the *CUST* and the *SUPP* matrices, whose elements are computed as follows:

11 $${CUST}_{ij}=\frac{{REVSHARE}_{ij}}{{OUTPUT}_{i}}$$

12 $${SUPP}_{ij}=\frac{{REVSHARE}_{ij}}{{INPUT}_{j}}$$

As shown in Eqs. (11) and (12), $${CUST}_{ij}$$ (the element in row $$i$$, column $$j$$ in the *CUST* matrix) shows the fraction of sector $$i$$’s revenue accounted for by sector $$j$$ and is obtained by dividing $${REVSHARE}_{ij}$$ by the total output value of sector *i* $${(OUTPUT}_{i}$$ in the *Make* table). $${SUPP}_{ij}$$ (the element in row $$i$$, column $$j$$ in the *SUPP* matrix) shows the fraction of sector $$j$$’s total inputs purchased from sector $$i$$ and is calculated by dividing $${REVSHARE}_{ij}$$ by the total value of sector $$j$$’s inputs ($${INPUT}_{j})$$.^14^ Thus, when considering a pair of sectors $$i$$ and $$j$$, we can compute a total of four business linkage measures: $${CUST}_{ji}$$, $${SUPP}_{ij}$$, $${CUST}_{ij}$$, and $${SUPP}_{ji}$$; they quantify the importance of the customer role of $$i$$ to $$j$$, the supplier role of $$i$$ to $$j$$, the customer role of $$j$$ to $$i$$, and the supplier role of $$j$$ to $$i$$, respectively. These variables will be employed as regressors in the cross-sectional regressions, where the regressants are the spillover measures from the variance decomposition matrix. The coefficients on these measures represent the impact of the trading relationship on the shock spillovers between sectors.

#### Summary statistics

**Fig. 5:**
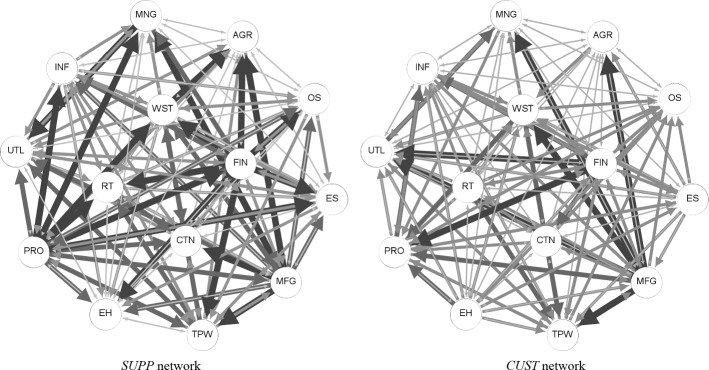
*SUPP* and *CUST* network. This figure shows the supplier and customer network of 14 sectors, using the average values of the *CUST* and *SUPP* variables during the whole sample period. The direction of the arrow shows the role of a sector in the trading relationship (i.e., a sector as a supplier / customer of the other sector). The thickness and colour of the arrow show the strength of the business linkage. A thick black arrow represents a relationship variable greater than 10%. A medium dark grey arrow represents a relationship variable between 5–10%. A thin grey arrow represents a relationship variable between 1–5%. The thinnest light grey arrow represents a relationship variable smaller than 1%

We construct the measures of economic linkages between pairs of sectors using the IO tables for each year and use in our analysis the average values over the sample period.^15^ Table 3 presents some summary statistics. For each sector, the columns labelled *Average* report the mean *SUPP* (*CUST*) values of a sector relative to all its partners over the sample period; they represent the average role of a sector as a supplier to (customer of) the other 13 sectors. We identify whether a sector serves as a main supplier or a main customer and report in the table the number of trading partners for which the *SUPP* and the *CUST* variables are larger than the thresholds of 1%, 5% and 10% (see also Horvath, 1998). A graphical representation of the supplier and customer network between sectors, using the average *SUPP* (*CUST*) values over the sample period, is provided in Fig. 5. The direction of an arrow shows the role of a sector as a supplier (customer) to its trading partner. The thickness and the shade of the arrow represent the strength of the relationship, based on the 1%, 5%, and 10% thresholds.

**Table 3:** Summary statistics of *CUST* and *SUPP* variables

Ector	SUPP				CUST				Either SUPP or CUST			Size	
	Average (%)	> 1%	> 5%	> 10%	Average (%)	> 1%	> 5%	> 10%	> 1%	> 5%	> 10%	Number of firms	Number of establishments
AGR	0.45	1	0	0	0.48	1	0	0	2	0	0	21,898	22,598
MNG	1.63	2	2	1	0.50	3	0	0	4	2	1	20,881	27,269
UTL	1.29	7	0	0	0.96	2	1	0	7	1	0	6,003	17,759
CTN	0.89	3	0	0	1.79	7	0	0	9	0	0	704,448	716,816
MFG	9.54	13	10	4	12.89	11	6	5	13	11	6	263,107	305,279
WST	3.40	12	3	1	1.80	6	2	0	13	5	1	315,473	419,001
RT	1.21	4	1	0	1.96	7	1	0	9	2	0	672,049	1,080,173
TPW	3.05	11	3	0	1.09	6	0	0	12	3	0	173,353	220,404
INF	2.15	11	0	0	1.17	6	0	0	12	0	0	74,065	141,497
FIN	9.29	13	10	7	4.43	10	4	2	13	12	9	532,533	852,665
PRO	9.75	13	12	6	2.45	8	1	0	13	12	6	1,141,406	1,315,263
EH	0.28	0	0	0	2.34	9	1	0	9	1	0	719,966	924,238
ES	1.15	6	0	0	1.55	9	0	0	10	0	0	616,988	791,868
OS	0.81	4	0	0	0.58	2	0	0	5	0	0	675,959	739,945

This table presents summary statistics for the 14 sectors. The *CUST* and *SUPP* variables are calculated using the average IO values over the sample period. The columns labelled Average *SUPP* / *CUST* report the average roles of a sector as a supplier / customer to the other sectors. The 1%, 5% and 10% threshold columns show the number of trading partners for which the *SUPP / CUST* or either *SUPP* or *CUST* value is greater than the corresponding thresholds. The last two columns show the average number of firms and number of establishments over the period 2005–2018

Considering the supplier role of a sector, according to Table 3 and Fig. 5, three sectors stand out: Professional and business services (PRO), Manufacturing (MFG), and Finance, insurance, real estate, rental and leasing (FIN) supply, on average, 9.75%, 9.54% and 9.29% of inputs to other sectors, respectively. Their dominant position in the supplier network is also confirmed by the number of sectors they serve as main suppliers: each of these three sectors provides over 1% of the inputs of each of the other 13 sectors, more than 5% of the inputs of 10–12 sectors, and more than 10% of the inputs of 4–7 sectors.

The finding that a few sectors play a disproportionately important role as input suppliers to others supports the notion of the *first-order interconnections* as described by Acemoglu et al. (2012). In their theoretical model, shocks to a dominant sector are shown to generate strong spillovers to direct customer sectors, leading to large aggregate volatility. Our earlier variance decomposition results are in line with Acemoglu et al. (2012). According to the estimated measures of directional spillovers, Professional and business services (PRO), Manufacturing (MFG) and Finance, insurance, real estate, rental and leasing (FIN) are found to be net shock transmitters. Importantly, the estimated net spillovers for these three sectors (PRO, MFG and FIN) are relatively large compared to the values for the other net shock transmitters.

On the other side of the coin, Educational services, health care, and social assistance (EH), Agriculture, forestry, fishing, and hunting (AGR), and Other services, except government (OS) are the three sectors that account for the smallest proportion of other sectors’ inputs, supplying only 0.28%, 0.45% and 0.81% of inputs to other sectors, respectively. While the latter two (AGR and OS) are among the sectors with the smallest outputs in the economy, Educational services, health care, and social assistance (EH) is the second largest supplier to final users, and has the largest proportion of its revenue accounted for by personal consumption. Consistent with the findings reported in Table 3 and Fig. 5, the results for the variance decomposition matrix shown in Table 2 show that these three industries are net shock receivers.

In what regards the customer role, the descriptive statistics in Table 3 designate Manufacturing (MFG) as the largest customer sector, accounting on average for 12.89% of the other sectors’ revenue. This sector contributes to more than 1% of the sales of 11 (out of 13) sectors, and to more than 10% of the revenues of 5 other sectors, as also illustrated by the number of thick arrows in Fig. 5. Once again, the importance of MFG in the supplier-customer network is consistent with its output magnitude and key role in a modern economy. Finance, insurance, real estate, rental and leasing (FIN) ranks as the second largest customer, with the average *CUST* value of 4.43%. Mining (MNG) and Agriculture, forestry, fishing, and hunting (AGR) are the smallest customers. A relative comparison of the average values of the *CUST* and *SUPP* variables together with the numbers of main customers and suppliers show that US sectors tend to have slightly more diversified customers than suppliers.

Table 3 also reports the number of trading partners with which a sector has strong linkages—either in terms of *CUST* or *SUPP—*at the 1%, 5% and 10% thresholds, respectively. These numbers show the closeness of a sector to other sectors in the network regardless of its role. Manufacturing (MFG), Wholesale trade (WST), Professional and business services (PRO) and Finance, insurance, real estate, rental and leasing (FIN) are the central sectors in the economy. Each of these sectors has strong trade linkages (higher than 1%) with all their trading partners (i.e., 13 other sectors). Agriculture, forestry, fishing, and hunting (AGR) is the least-connected sector; it is closely related to two other sectors at the 1% threshold, and has no close linkage at the 5% and 10% thresholds.

The last two columns of Table 3 report information about sector size: the number of firms and the number of establishments. This data is obtained from the US Census Bureau’s Statistics of US Businesses (SUSB).^16^ Utilities (UTL) is the smallest sector, with 6,003 firms and 17,759 establishments, whereas Professional and business services (PRO) is the largest sector, with 1,141,406 firms and 1,315,263 establishments.

### Pairwise spillovers and business linkages

For each pair of sectors $i$ and $j$, we obtain two directional spillover measures showing the fraction of the forecast error variance of sector $i$ which is due to shocks to sector $j$, denoted by ${S}_{i\leftarrow j}^{H}$, and the fraction of the forecast error variance of sector $j$ which is due to shocks to sector $i$, denoted by ${S}_{j\leftarrow i}^{H}$*.* These measures are the ${ij}^{th}$ and the ${ji}^{th}$ elements in the variance decomposition matrix shown in Table 2 and are employed as the dependent variable in the cross-sectional regression model specified in Eq. (13) below.

13 $${S}_{i\leftarrow j}^{H}={\alpha }_{0}+{\alpha }_{1}{CUST}_{ij}+{\alpha }_{2}{SUPP}_{ji}+{\alpha }_{3}{CUST}_{ji}+{\alpha }_{4}{SUPP}_{ij}+{\alpha }_{5}{Size}_{j}+{\alpha }_{6}{Size}_{i}+{u}_{ij}$$

where ${S}_{i\leftarrow j}^{H}$ is the pairwise spillover from sector $j$ to sector $i$, showing the proportion of the $H$-step-ahead forecast error variances of sector $i$ accounted for by shocks to sector $j$. The first four regressors—${{CUST}_{ij}, { SUPP}_{ji}, CUST}_{ji},$ and ${SUPP}_{ij}$—are the business linkage variables constructed from the IO accounts. They quantify the importance of a sector as a supplier or a customer for its trading partner. ${SUPP}_{ij}$ shows the supplier role of sector $i$ to sector $j$, while ${CUST}_{ij}$ shows the customer role of sector *j* to sector $i$*.*${SUPP}_{ji}$ and ${CUST}_{ji}$ are defined similarly*.* In line with Ahern (2013), we control also for the impact of the sector size on shock spillovers. We expect a larger sector, with a large number of constituent firms, to have stronger influence on other sectors in the economy. We construct 91 trading pairs from the 14 sectors in our sample, giving us 182 pairwise directional spillover observations. Note that because the value for the dependent variable is obtained from the variance decomposition matrix, we follow Lewis and Linzer (2005) and Weiß et al. (2014) to estimate and report the heteroskedasticity-consistent standard errors as discussed in White (1980).

Table 4 reports the estimates of Eq. (13). We find that the degree of pairwise spillovers from sector $j$ to sector $i$ is positively related to the supplier share of sector $j$ in the total input of sector $i$. In other words, shocks to an important supplier sector contribute strongly to its partner’s forecast error variance. This finding highlights the importance of the supplier role for intersectoral shock spillovers. It may be attributed (partly) to the high aggregation at the sectoral level, which reduces considerably the possibility of substituting input suppliers. Our results are in line with the findings in Barrot and Sauvagnat (2016) who document the negative impact on a firm’s sales growth following input disruption (caused by a natural disaster) to the firm’s supplier.

**Table 4:** Pairwise spillovers and business linkages in 2005–2020

	Pairwise spillover	
	(1)	(2)
Customer role of sector (*CUST*_*ij*_)	0.025	0.026
	(0.024)	(0.023)
Supplier role sector (*SUPP*_*ji*_)	0.156***	0.154***
	(0.025)	(0.025)
Customer role of partner (*CUST*_*ji*_)	− 0.016	− 0.015
	(0.019)	(0.019)
Supplier role partner (*SUPP*_*ij*_)	0.024	0.022
	(0.024)	(0.024)
Sector's number of firms	0.013***	
	(0.003)	
Partner's number of firms	0.005*	
	(0.003)	
Sector's number of establishments		0.010***
		(0.002)
Partner's number of establishments		0.004*
		(0.002)
Observations	182	182
Adjusted R-squared	0.335	0.332

This table reports the cross-sectional estimated coefficients and robust standard errors (in parentheses) of the regression specified in Eq. (13). The dependent variable is the pairwise spillover obtained from the generalized variance decomposition approach over the period 3 January 2005–31 December 2020. The business linkage variables and sector size (millions of firms/establishments) are the average values during the sample period. *, ** and *** denote the 10%, 5% and 1% significance levels, respectively

The size of the trading sectors appears to positively correlate with the pairwise shock spillovers as the coefficients associated with the number of firms (column 1) and the number of establishments (column 2) are positive and statistically significant. This suggests that a sector which has a large number of firms or establishments, with an extended trading network with other sectors, tends to easily transmit and receive shocks to/ from other sectors. This finding is in line with Herskovic et al. (2020), which confirms the size impacts on volatility transmission at firm level.

### Total directional spillovers and business linkages

We now investigate whether total directional spillovers are affected by the role a sector plays as either a supplier or a customer. Table 5 reports the results for the total directional spillover from a sector TO others (columns 1–3), FROM others to a sector (columns 4–6), and the NET total directional spillover (columns 7–9). The explanatory variable is the number of trading partners with close business linkages between a sector and the others regardless of its role (i.e., whether supplier or customer to the other sectors), as reported in the last three columns of Table 3 at various thresholds. The results reported in Table 5 reveal a positive significant relationship between the total directional spillovers and the number of close economic linkages at all three thresholds: 1%, 5% and 10%. Our findings are in line with Carvalho (2014), who demonstrates that hub sectors, which are close to the majority of other sectors, comove more with others. Ahern (2013) also indicates that shocks to sectors which are central in the network are more likely to contribute more to the aggregate system-wide volatility. Given the small sample size, however, these findings should be interpreted with care.

**Table 5:** Total directional spillovers and business linkages in 2005–2020

	From sector TO others			FROM others to sector			Net Total Directional Spillover		
	(1)	(2)	(3)	(4)	(5)	(6)	(7)	(8)	(9)
Number of either *CUST* or *SUPP* > 1%	0.047***			0.007***			0.040***		
	(0.006)			(0.001)			(0.005)		
Number of either *CUST* or *SUPP* > 5%		0.027***			0.003***			0.023***	
		(0.008)			(0.001)			(0.007)	
Number of either*CUST* or *SUPP* > 10%			0.032**			0.004**			0.028**
			(0.015)			(0.002)			(0.014)
Observations	14	14	14	14	14	14	14	14	14
Adjusted R-squared	0.810	0.374	0.194	0.827	0.237	0.107	0.799	0.395	0.208

This table reports the cross-sectional estimated coefficients and robust standard errors (in parentheses) of the impact of business linkages on the directional spillovers obtained from the generalized variance decomposition approach over the period 3 January 2005–31 December 2020. The dependent variables are the directional spillovers from a sector TO others (columns (1)–(3)), the directional spillovers FROM others to a sector (columns (4)–(6)) and the net total directional spillovers (columns (7)–(9)). The number of either *SUPP* or *CUST* > 1%, 5% and 10% show the number of trading partners that either the *SUPP* or *CUST* values of the sector (calculated as the average of the values from 2005–2019 IO tables) are greater than the corresponding thresholds. *, ** and *** denote the 10%, 5% and 1% significance levels, respectively

**Part III: Extensions and robustness tests**

## Market conditions

Our 16-year sample period spans different market conditions. In this section, we examine the impact of economic relationship on shock transmissions during the sub-periods: the pre-crisis 2005–2006, the financial crisis 2007–2008, the post-crisis 2009–2019, and the COVID-19 pandemic 2020. Specifically, we fit the VARX model for 14 sectors in each sub-period and calculate the spillover measures using the generalized forecast variance approach. We then regress these measures on the average business linkage variables calculated for each sub-period. Panels A-D of Table 6 show the error variance decomposition matrices computed using data for the sub-periods. We immediately notice that the total spillovers increase from 82.32% in the pre-crisis period to 88% in the crisis and 84.31% in the post-crisis period. The lowest total directional spillover from a sector TO others is 38.37% (Mining—MNG) in Panel A, which is considerably smaller than 61.74% (Mining—MNG) in Panel B, and 47.8% (Agriculture, forestry, fishing, and hunting—AGR) in Panel C. The lowest total directional spillover FROM others to a sector also increases from 69.83% (Mining—MNG) in the pre-crisis period to 83.62% (Mining—MNG) and 76.81% (Agriculture, forestry, fishing, and hunting—AGR) in the crisis and post-crisis period, respectively. In terms of pairwise spillover, the smallest cross-variance share is 1.23% from Mining (MNG) to Retail trade (RT) in the sub-periods 2005–2006, 2.96% from Mining (MNG) to Finance, insurance, real estate, rental and leasing (FIN) in 2007–2008, and 3.42% from Agriculture, forestry, fishing, and hunting (AGR) to Mining (MNG) and to Utility (UTL) in the sub-period 2009–2019. Most net spillovers declined relative to their values prior to the financial crisis. This is true for both the largest net shock receivers and transmitters. Overall, our findings confirm stronger shock spillovers between sectors during and after the financial crisis.

**Table 6:** Shock spillover—separate samples

	AGR	MNG	UTL	CTN	MFG	WST	RT	TPW	INF	FIN	PRO	EH	ES	OS	FROM others
*Panel A: shock spillover—pre-crisis 2005–2006*															
AGR	22.25	4.27	4.07	7.33	8.53	7.74	5.67	6.50	5.75	6.85	8.04	3.68	5.64	3.68	77.75
MNG	5.44	30.17	9.17	7.05	10.51	6.76	2.30	6.77	3.10	3.47	6.94	2.65	2.39	3.27	69.83
UTL	3.74	6.42	20.79	5.46	9.25	7.64	4.79	5.35	5.77	8.09	8.47	5.24	4.92	4.07	79.21
CTN	5.56	4.10	4.56	17.60	8.70	8.76	8.22	7.16	4.91	7.28	8.49	4.41	5.79	4.46	82.40
MFG	4.72	4.27	5.46	6.09	12.26	9.25	7.11	7.53	7.94	8.64	10.01	5.23	6.90	4.59	87.74
WST	4.58	2.91	4.86	6.57	9.99	13.20	7.65	7.88	6.89	8.48	9.74	5.89	6.83	4.54	86.80
RT	3.94	1.23	3.53	7.14	8.89	8.85	15.28	7.31	7.09	9.18	9.01	5.02	8.65	4.86	84.72
TPW	4.58	3.55	4.06	6.43	9.74	9.41	7.54	15.83	6.26	7.90	9.48	4.21	6.83	4.18	84.17
INF	4.28	1.67	4.52	4.50	10.52	8.38	7.46	6.37	16.16	9.18	10.28	4.44	7.60	4.63	83.84
FIN	4.32	1.64	5.46	5.67	9.72	8.80	8.20	6.82	7.75	13.76	9.75	5.81	7.68	4.61	86.24
PRO	4.51	2.85	5.06	6.07	10.14	9.18	7.32	7.48	7.89	8.80	12.42	5.52	7.65	5.10	87.58
EH	3.40	1.84	5.19	5.08	8.68	9.05	6.61	5.22	5.49	8.28	9.04	20.24	6.66	5.23	79.76
ES	4.10	1.27	3.72	5.31	8.98	8.28	9.16	7.15	7.39	8.87	9.82	5.18	15.94	4.83	84.06
OS	3.57	2.33	4.24	5.61	8.11	7.48	6.92	5.73	6.08	7.24	8.92	5.56	6.55	21.66	78.34
TO others	56.72	38.37	63.91	78.33	121.75	109.57	88.95	87.28	82.30	102.25	117.98	62.84	84.11	58.05	**82.32**
NET	− 21.03	− 31.46	− 15.31	− 4.07	34.01	22.77	4.24	3.11	− 1.54	16.02	30.41	− 16.92	0.06	− 20.29	

	AGR	MNG	UTL	CTN	MFG	WST	RT	TPW	INF	FIN	PRO	EH	ES	OS	FROM others
*Panel B: Shock spillover—financial crisis 2007–2008*															
AGR	14.58	4.20	4.81	6.83	7.73	7.56	7.37	7.08	6.81	6.63	7.75	5.40	7.06	6.20	85.42
MNG	4.66	16.38	8.39	5.85	10.57	7.78	4.55	6.68	7.42	3.91	8.08	5.49	5.22	5.02	83.62
UTL	4.31	7.27	13.60	4.08	9.86	8.02	6.22	6.74	8.56	4.81	8.34	6.58	6.12	5.51	86.40
CTN	5.85	4.57	3.84	12.54	7.45	7.51	7.84	8.12	6.65	8.24	8.50	4.81	7.71	6.38	87.46
MFG	5.03	6.83	7.16	5.67	10.04	8.41	6.91	7.36	8.70	6.02	8.81	6.24	6.98	5.84	89.96
WST	5.16	5.22	6.22	5.94	8.92	10.08	7.53	7.50	8.12	6.34	8.73	6.35	7.12	6.79	89.92
RT	5.45	3.53	5.29	6.75	8.06	8.24	10.79	7.79	7.79	7.20	8.42	6.03	8.32	6.34	89.21
TPW	5.16	4.87	5.64	7.05	8.46	8.16	7.71	11.05	7.55	6.71	8.51	5.59	7.79	5.75	88.95
INF	4.82	5.40	6.81	5.41	9.48	8.29	7.27	7.17	10.42	6.44	9.04	6.25	7.39	5.80	89.58
FIN	5.36	2.96	4.29	7.91	7.53	7.57	7.94	7.34	7.53	12.03	9.13	5.82	7.99	6.61	87.97
PRO	4.97	4.97	5.92	6.57	8.71	8.28	7.34	7.46	8.33	7.36	9.86	6.43	7.36	6.46	90.14
EH	4.56	4.28	6.17	4.96	8.24	8.13	7.05	6.58	7.75	6.30	8.62	13.06	7.25	7.02	86.94
ES	5.23	3.79	5.15	6.71	8.09	7.83	8.41	7.85	7.93	7.26	8.39	6.16	10.87	6.32	89.13
OS	5.22	3.86	5.02	6.47	7.61	8.48	7.36	6.61	7.09	6.95	8.42	6.85	7.37	12.71	87.29
TO others	65.78	61.74	74.71	80.21	110.71	104.25	93.50	94.27	100.23	84.16	110.74	77.99	93.68	80.03	**88.00**
NET	− 19.64	− 21.88	− 11.69	− 7.25	20.75	14.32	4.29	5.31	10.65	− 3.82	20.60	− 8.95	4.55	− 7.26	

	AGR	MNG	UTL	CTN	MFG	WST	RT	TPW	INF	FIN	PRO	EH	ES	OS	FROM others
*Panel C: shock spillover—post crisis 2009–2019*															
AGR	23.19	4.15	3.45	6.31	7.26	6.86	5.63	6.63	6.05	6.85	7.66	4.65	5.88	5.43	76.81
MNG	3.42	19.22	4.12	6.81	9.31	7.00	5.14	7.84	6.43	7.05	7.77	4.46	6.55	4.88	80.78
UTL	3.42	4.94	23.08	5.32	8.81	8.79	6.12	6.51	6.58	5.40	7.06	4.43	5.05	4.50	76.92
CTN	4.11	5.35	3.45	15.28	8.44	7.69	6.78	7.81	6.86	7.69	8.49	5.17	7.03	5.85	84.72
MFG	3.55	5.51	4.34	6.33	11.36	9.01	7.77	7.82	8.87	7.06	9.45	5.70	7.55	5.68	88.64
WST	3.74	4.65	4.85	6.45	10.04	12.68	7.92	7.98	7.86	6.92	8.72	5.36	7.02	5.81	87.32
RT	3.36	3.74	3.71	6.23	9.50	8.70	13.88	7.38	9.24	6.13	8.77	5.51	7.97	5.89	86.12
TPW	3.83	5.48	3.78	6.93	9.24	8.47	7.14	13.41	7.44	7.54	8.51	4.97	7.71	5.55	86.59
INF	3.34	4.34	3.67	5.84	10.10	8.02	8.59	7.15	12.94	6.79	10.21	5.23	8.09	5.68	87.06
FIN	4.20	5.23	3.35	7.27	8.76	7.81	6.26	7.94	7.45	14.36	10.22	4.84	6.87	5.45	85.64
PRO	3.86	4.74	3.58	6.55	9.73	8.08	7.40	7.41	9.23	8.44	11.75	5.67	7.55	6.02	88.25
EH	3.55	4.12	3.43	6.06	8.94	7.55	7.07	6.58	7.18	6.04	8.61	17.81	6.59	6.46	82.19
ES	3.52	4.74	3.02	6.46	9.25	7.72	7.96	7.99	8.71	6.73	8.98	5.15	13.95	5.83	86.05
OS	3.90	4.24	3.24	6.41	8.36	7.68	7.09	6.89	7.35	6.43	8.60	6.08	7.00	16.71	83.29
TO others	47.80	61.23	47.98	82.98	117.74	103.38	90.87	95.92	99.27	89.07	113.06	67.22	90.84	73.04	**84.31**
NET	− 29.01	− 19.56	− 28.95	− 1.74	29.10	16.06	4.75	9.33	12.21	3.42	24.80	− 14.97	4.80	− 10.25	

	AGR	MNG	UTL	CTN	MFG	WST	RT	TPW	INF	FIN	PRO	EH	ES	OS	FROM others
*Panel D: shock spillover—COVID-19 pandemic 2020*															
AGR	21.38	5.00	4.29	5.48	6.63	6.71	3.64	7.55	5.34	8.78	7.91	6.17	5.56	5.56	78.62
MNG	4.04	16.74	4.12	6.48	7.00	8.13	2.52	9.51	3.99	9.53	7.71	6.95	6.80	6.48	83.26
UTL	4.10	3.21	17.16	5.45	9.49	8.82	5.16	6.51	6.24	9.33	9.28	6.60	3.01	5.63	82.84
CTN	3.90	5.40	4.75	14.19	7.13	7.31	3.34	8.83	4.81	8.20	8.45	8.84	7.35	7.50	85.81
MFG	3.50	3.99	6.24	5.19	11.88	8.69	7.91	7.16	9.84	8.18	10.08	7.14	4.43	5.78	88.12
WST	3.88	5.20	6.28	5.88	9.45	11.73	4.85	8.40	6.62	9.54	9.45	6.74	5.17	6.81	88.27
RT	2.88	2.25	4.91	4.05	12.33	6.83	17.25	5.46	14.03	6.23	9.45	6.21	3.54	4.57	82.75
TPW	4.19	6.33	5.09	7.13	8.12	8.67	3.78	11.69	5.64	9.30	8.71	7.40	7.67	6.28	88.31
INF	3.31	2.91	4.91	4.12	11.79	7.44	10.57	6.21	14.15	7.25	10.80	6.80	4.37	5.37	85.85
FIN	4.78	5.96	6.40	6.21	8.22	9.04	3.87	8.47	5.64	12.15	8.81	7.22	5.98	7.25	87.85
PRO	4.04	4.44	5.97	5.95	9.76	8.51	5.75	7.60	8.64	8.58	11.16	7.34	5.53	6.73	88.84
EH	3.65	5.08	4.90	7.65	8.25	7.24	4.15	7.97	6.30	8.47	8.85	12.82	7.48	7.21	87.18
ES	4.19	5.92	3.39	7.71	6.30	7.09	3.19	9.86	4.86	8.39	7.99	8.80	14.52	7.78	85.48
OS	3.86	5.06	4.90	6.90	7.48	8.14	3.72	7.27	5.92	9.12	9.02	7.72	7.16	13.74	86.26
TO others	50.30	60.74	66.16	78.18	111.94	102.61	62.46	100.81	87.88	110.89	116.51	93.93	74.05	82.96	**85.67**
NET	− 28.32	− 22.51	− 16.68	− 7.63	23.83	14.34	− 20.29	12.50	2.03	23.04	27.67	6.76	− 11.43	− 3.30	

This table reports shock spillovers between 14 sectors over sub-sample periods.

Panel A: The sub-sample period 3 January 2005 – 31 December 2006

Panel B: The sub-sample period 2 January 2007 – 31 December 2008

Panel C: The sub-sample period 2 January 2009 – 31 December 2019

Panel D: The sub-sample period 2 January 2020 – 31 December 2020

The $ij$*-*th element of the upper-left $14\times 14$ submatrix reports the $ij$*-*th pairwise spillover in percentage, i.e., the fraction of 10-day-ahead forecast error variance of sector $i$ accounted for by shocks to sector $j$*.* The rightmost column, FROM others, shows the total directional spillovers from all other sectors to a sector $i$ (sum of off-diagonal entries in row $i$). The bottom row, TO others, shows the total directional spillovers from sector $j$ to all other sectors (sum of off-diagonal entries in column $j$). The bottommost row (NET) shows the difference between the total directional spillovers TO and FROM other sectors to a specific sector. The bottom-right element (in boldface) gives the total spillovers between sectors

Panel D reveals the changes in sectoral shock spillovers due to the COVID-19 outbreak in 2020, which is consistent with our findings in the rolling window analysis. As compared to the spillovers in Panel C, the greatest change in the total directional spillovers, hence the greatest changes in the net spillovers, is a decrease of 28.41% in Retail Trade (RT). A closer look at the pairwise spillover shows that the largest changes in pairwise spillovers are also related to the Retail Trade (RT) sector: an increase of 4.79% in the shock spillover that Retail Trade (RT) receives from Information (INF) and a decrease of 4.78% in the shock spillover from Retail Trade (RT) to Arts, entertainment, recreation, accommodation, and food services (ES). The fact that Retail trade (RT) changes from a shock transmitter to a shock receiver in the system reflects how COVID-19 affects business operations. Retail businesses have been hardly hit by lockdown and social distancing measures implemented in many countries, including the US. Thus, it is understandable why Retail trade (RT) becomes less influential and more prone to shock spillovers. In the same vein, Arts, entertainment, recreation, accommodation, and food services (ES) also suffers a decline in shock transmission, with a decrease of 1.29% of pairwise spillover to others on average. Finally, due to the pandemic, pairwise shock spillovers from Educational services, health care, and social assistance (EH) to every other sector increase by an average of 2.06%, resulting in an increase of 26.72% in the total directional spillover TO others, changing it from a shock receiver to a shock transmitter.

Tables 7 and 8 report the results of the extended analysis in different market conditions.^17^ All coefficients representing the impacts of the supplier/customer roles on intersectoral shock transmissions preserve their signs and significance across the three sub-periods. The magnitude of the estimates on the trade linkage variables weakens slightly during the crisis period, but recovers in the post-crisis period. Similar to Ahern (2013), we note that the economic linkages are not the only channel for shock transmissions between sectors. In addition to their connectedness in the production network, sectors connect to each other via complicated links of their constituent firms, in terms of various relationships such as ownership and geographic closeness. Thus, in periods of stress, shock spillovers may be more strongly affected by other relationships than their economic linkages. However, even though the impacts of the business linkages weaken in the crisis period, our results clearly demonstrate that they invariably matter for intersectoral shock transmissions in all market conditions.

**Table 7:** Pairwise spillovers and business linkages—separate samples

	2005–2006		2007–2008		2009–2019	
	(1)	(2)	(3)	(4)	(5)	(6)
Customer role of sector (*CUST*_*ij*_)	0.048	0.051	0.041*	0.042*	0.021*	0.023*
	(0.032)	(0.032)	(0.023)	(0.023)	(0.025)	(0.025)
Supplier role sector (*SUPP*_*ji*_)	0.230***	0.223***	0.100***	0.097***	0.176***	0.173***
	(0.038)	(0.037)	(0.023)	(0.023)	(0.027)	(0.028)
Customer role of partner (*CUST*_*ji*_)	− 0.018	− 0.014	0.007	0.008	− 0.025	− 0.022
	(0.024)	(0.024)	(0.019)	(0.019)	(0.020)	(0.020)
Supplier role partner (*SUPP*_*ij*_)	0.063	0.059	0.014	0.012	0.034	0.032
	(0.038)	(0.038)	(0.027)	(0.026)	(0.028)	(0.028)
Sector's number of firms	0.016***		0.010***		0.014***	
	(0.003)		(0.003)		(0.003)	
Partner's number of firms	0.007*		0.004		0.006	
	(0.004)		(0.003)		(0.003)	
Sector's number of establishments		0.015***		0.009***		0.012***
		(0.003)		(0.002)		(0.003)
Partner's number of establishments		0.007**		0.003		0.005
		(0.003)		(0.002)		(0.003)
Observations	182	182	182	182	182	182
Adjusted R-squared	0.391	0.402	0.253	0.256	0.310	0.312

This table reports the cross-sectional estimated coefficients and robust standard errors (in parentheses) of the regression specified in Eq. (13) in three sub-samples. The dependent variable is the pairwise directional spillover measures obtained from the generalized variance decomposition approach over each sub-sample period. The business linkage variables and sector size (millions of firms / establishments) are the average values during the sub-sample period. *, ** and *** denote the 10%, 5% and 1% significance levels, respectively

**Table 8:** Total directional spillovers and business linkages—separate samples

	From sector TO others			FROM others to sector			Net total directional spillover		
	(1)	(2)	(3)	(4)	(5)	(6)	(7)	(8)	(9)
*Panel A: Sample period 2005–2006*									
No *CUST* or *SUPP* > 1%	0.056***			0.011***			0.044***		
	(0.008)			(0.002)			(0.006)		
No *CUST* or *SUPP* > 5%		0.041***			0.006***			0.035***	
		(0.007)			(0.001)			(0.006)	
No *CUST* or *SUPP* > 10%			0.049***			0.007***			0.041***
			(0.016)			(0.002)			(0.014)
Adjusted R-squared	0.772	0.509	0.269	0.818	0.257	0.110	0.733	0.560	0.303
*Panel B: Sample period 2007–2008*									
No *CUST* or *SUPP* > 1%	0.034***			0.004***			0.030***		
	(0.005)			(0.001)			(0.004)		
No *CUST* or *SUPP* > 5%		0.017**			0.002**			0.016**	
		(0.008)			(0.001)			(0.008)	
No *CUST* or *SUPP* > 10%			0.019			0.002			0.017
			(0.015)			(0.001)			(0.014)
Adjusted R-squared	0.736	0.192	0.033	0.768	0.088	− 0.021	0.722	0.206	0.041
*Panel C: Sample period 2009–2019*									
No *CUST* or *SUPP* > 1%	0.053***			0.009***			0.044***		
	(0.007)			(0.001)			(0.006)		
No *CUST* or *SUPP* > 5%		0.029***			0.004***			0.025***	
		(0.009)			(0.001)			(0.008)	
No *CUST* or *SUPP* > 10%			0.035**			0.005**			0.030**
			(0.018)			(0.002)			(0.015)
Adjusted R-squared	0.687	0.316	0.162	0.638	0.203	0.095	0.688	0.337	0.175

This table reports the cross-sectional estimated coefficients and robust standard errors (in parentheses) of the impact of business linkages on the directional spillovers obtained from the generalized variance decomposition approach over three sub-sample periods. The dependent variables are the directional spillovers from a sector TO others (columns (1)–(3)), the directional spillovers FROM others to a sector (columns (4)–(6)) and the net total directional spillovers (columns (7)–(9)). The number of either *SUPP* or *CUST* > 1%, 5% and 10% show the number of trading partners that either the *SUPP* or *CUST* values of the sector (calculated as the average of the values of IO tables in each sub-period) are greater than the corresponding thresholds. *, ** and *** denote the 10%, 5% and 1% significance levels, respectively

## Robustness checks

This section collects a series of sensitivity tests to check for the robustness of our results.

*Non-financial sectors****.*** Regardless of its actual trading relationship with the other sectors, the financial sector is more sensitive to shocks from others and more likely to transmit significant shocks to other sectors. To ensure that our results are not driven by it, we drop the Finance, insurance, real estate, rental, and leasing (FIN) sector from our sample. We confirm the importance of the business linkages on volatility spillovers between non-financial sectors (results available upon request).

*Forecast horizon and VARX order.* We check the sensitivity of our results to the choice of forecast horizon and VARX order. We estimate the model in Eq. (13) using the spillover measures obtained from the VARX specification of orders from one to four as well as the forecast horizons of 1 to 5 days and 20 days. We obtain qualitatively similar results (available upon request) to those reported in Tables 4 and 5.

*Alternative business-linkage variables.* Finally, we check whether our cross-sectional regression results are robust to the choice of the IO tables. In addition to using the average values of the business linkage variables over the full sample period, we use the variables calculated from the IO tables of every year from 2005 to 2019. The unreported results are qualitatively similar to our main findings, confirming that the impact of business linkages on shock spillovers between sectors persists regardless of the choice of year for the IO tables.

## Conclusions

We investigate the degree of financial connectedness as well as the characteristics of shock transmissions among the 14 US economic sectors using a VARX system based on the error variance decomposition approach proposed by Diebold and Yilmaz (2012, 2014). The total shock spillover among all sectors is found to be considerably large (85.8%), pointing to significant financial connectedness among the sectors. We find that, on average, shocks to a sector’s return can explain 6.6% of the forecast error variance of its trading partner. Manufacturing (MFG), Professional and business services (PRO), and Wholesale Trade (WST) are the top three net shock transmitters, while Agriculture, forestry, fishing, and hunting (AGR) and Mining (MNG) are the smallest shock transmitters. Our dynamic analysis using 200-day rolling windows and the analysis on subsamples show evidence of stronger intersectoral shocks spillovers in turbulent markets during the 2007–2008 Financial Crisis and the COVID-19 pandemic. We observe an increase in the total directional shock spillovers from Educational services, health care, and social assistance (EH) to other sectors, while Retail trade (RT) becomes more sensitive to shock spillovers, changing from a shock transmitter to a shock receiver. This evidence reflect the impact of COVID-19 on different sectors of the economy.

We also examine the relevance of supplier-customer linkages on the stock return spillovers across sectors. We uncover a strong relation between supplier-customer linkages and shock transmissions between US sectors: shock spillovers from a sector to its partner are significant and can be explained by the supplier role of the sector. In addition, we find that the total directional spillovers from / to a sector are affected by the closeness of the sector to others and that the number of close trading partners of a sector with other sectors is positively related to the net shock spillover which the sector transmits to others.

The robustness of our results is confirmed under different settings. We obtain qualitatively similar results when the analysis excludes the financial sector from the sample. Our results are robust to the use of business linkage variables calculated using information from the IO tables for different years. They are also insensitive to the order of the VARX model and the choice of forecast horizon.

Our findings are useful for investors whose portfolios concentrate in some specific sectors or industries. While sector funds are more manageable and shown to deliver outperformance, they entail greater total and systematic risk. Thus, our evidence of sectoral shock transmission taking into account business linkages provides practical implications for risk management. Investors can observe the shocks to the sector’s major suppliers to better predict the volatility of their positions. Moreover, our findings are useful for policy makers and regulators, since understanding how shocks to a sector can affect others, ultimately resulting in aggregate fluctuation, is crucial, especially in periods of stress such as the financial crisis or the COVID-19 pandemic.

Our work is not without limitations. Our first stage results would likely be affected if investors take various business horizons into consideration. One fruitful direction for future research thus relates to the rich time–frequency dynamics of volatility connectedness in the spirit of Barunik and Krehlik (2018). We have not pursued this avenue as the approach does not match the frequency of the supplier-customer measures employed in our second stage analysis. We therefore leave this unexplored area to future researchers.

## Supplementary Information

Below is the link to the electronic supplementary material.

Supplementary file1 (DOCX 48 kb)

## Data Availability

Available upon request.

## Footnotes

1 They argue that an economy in which a small number of sectors play a disproportionately large role as input suppliers to other sectors is likely to experience large output fluctuation. See also Long and Plosser (1983), Horvath (1998), and Shea (2002).

2 Other estimates of the contribution of sectoral shocks to the aggregate US output variation are provided by Foerster et al. (2011) and Ando (2014).

3 Diebold and Yilmaz (2014) demonstrate that their approach is intimately related to both modern network theory and modern measures of systemic risk. They regard the variance decomposition matrix as a network adjacency matrix with each element in the matrix representing the strength of the connectedness. This method has been used to model risk and return spillovers of stock markets (Yarovaya et al., 2016), sovereign credit markets (Alter and Beyer, 2014; Claeys and Vašíček, 2014), traditional currencies and/or cryptocurrencies (Antonakakis, 2012; Ji et al., 2019), etc.

4 The advantange of this approach relies in its ability to model shock spillovers among *all* the sectors in the system as opposed to modelling spillovers between pairs of sectors in isolation of the other sectors, as feasible in a multivariate GARCH approach (see, for instance, Nguyen et al., 2020).

5 It is worth noting that Barunik and Krehlik (2018) propose a framework which allows the analysis of connectedness among sectors based on heterogenous frequency responses to shocks. While their approach has its own merit, we do not employ it in this paper as it does not allow us to conduct our second stage investigation of the impact of business linkages on shock spillovers.

6 CRSP does not provide NAICS codes before 2005.

7 We estimate four VARX specifications of orders from 1 to 4. The first-order VARX is the best specification satisfying three selection criteria: Bayesian Information Criterion (BIC), Akaike Information Criterion (AIC) and Hannan-Quinn Criterion (HQC). We report the results for 10-day forecast horizon, which is commonly used in related literature (see Diebold & Yilmaz, 2009; Antonakakis, 2012, among others). Section 7 shows that our results are not sensitive to the choice of VARX orders and predictive horizons.

8 We show later in the paper that the measures of connectedness in the variance decomposition matrix correlate positively with the measures of economic linkages between sectors. For instance, MFG is one of the largest suppliers of INF, while MNG is the smallest supplier of RT – see Appendix Table A6.

9 The value of a sector’s total directional spillover to others, by construction, can be larger than 100%.

10 However, Antonakakis (2012), Diebold and Yilmaz (2012), Claeys and Vašíček (2014) observe relatively smaller total spillovers.

11 Diebold and Yılmaz (2012) use 200-day rolling windows when examining a system of 13 financial institutions. A similar approach is used by Claeys and Vašíček (2014) and Yarovaya et al. (2016).

12 The pairwise spillovers between sectors are relevant in the rolling-sample windows as well and contribute to the dynamics of the above total measures. As our sample consists of 14 sectors, it is not feasible to present plots and detailed analysis for 182 pairwise and 91 net directional spillovers in this paper. Nonetheless, we do not disregard these pairwise directional spillovers. Indeed, the second part of our analysis focuses attention on the relationship between the pairwise spillovers and the strength of the business linkages between sectors.

13 For the above calculations, we assume that market shares are constant for every use of commodity. For example, if 60% of the total supply of commodity $c$ is produced by sector $i$ (i.e., *SHARE*_*ic*_ = 0.6), then 60% of the commodity $c$ that sector $j$ purchases as inputs for productions is attributed to sector $i$.

14 While the *Use* table records Labor (compensation of employees) as an input for production, there is no Labor sector in the *Make* table. Following Ahern and Harford (2014), we create an artificial Labor sector to ensure our calculations accurately account for the supply of this input. This sector will not be included in our final sample.

15 The *Average* is calculated for the period from 2005–2019 since data for IO 2020 is unavailable. Using instead the values from the 2007, 2012, 2017 benchmark tables, or from the other estimated tables leaves our findings unaltered. The statistics for the linkage variables calculated for every year are available upon request.

16 The numbers of firms / establishments reported are the average values over the 14-year period from 2005 to 2018 due to data availability.

17 As the data of 2020 IO tables are unavailable, the second stage investigation is performed for the three sub-samples: 2005–2006, 2007–2008 and 2009–2019.

## Code availability

Available upon request.
