# Supplementary material for: Shock transmissions and business linkages among US sectors
Source: Ann Oper Res. 2022 Dec 5:1–36. Online ahead of print. doi: 10.1007/s10479-022-04979-8 (PMC9734630; doi:10.1007/s10479-022-04979-8)
Supplement: Supplementary file 1 — Supplementary file1 (DOCX 48 kb) [file 10479_2022_4979_MOESM1_ESM.docx]

**Appendix - Input-Output Accounts and Constructed Tables**

**Table A1: MAKE Table (2012)**

This table is extracted from the *Make* table (2012), provided by the Bureau of Economic Analysis (BEA), showing the make of 17 commodities by 15 US sectors. Each sector is presented in a row and each commodity is shown in a column. The element in row $i$, column $c$ (${Make}_{ic}$) reports the value of commodity $c$ produced by sector $i$. The sum of all elements in a row is the Total Sector Output and the sum of all elements in a column is the Total Commodity Output.

(Millions of dollars)

| **Sectors / Commodities** | AGR | MNG | UTL | … | GOV | Used | Others | **Total Sector Output** |
| --- | --- | --- | --- | --- | --- | --- | --- | --- |
| AGR | 449,781 | - | - | … | - | - | - | 452,635 |
| MNG | - | 563,456 | - | … | - | - | - | 613,049 |
| UTL | - | 84 | 454,946 | … | 1,503 | - | - | 461,485 |
| … | … | … | … | … | … | … | … | … |
| ES | - | - | - | … | - | 131 | - | 1,093,441 |
| OS | - | - | - | … | - | - | - | 576,265 |
| GOV | 3,314 | 183 | 137,371 | … | 2,675,025 | 3,573 | 2,906 | 3,389,762 |
| **Total Commodity Output** | 454,461 | 567,943 | 592,434 | … | 2,676,528 | 8,542 | 2,906 | 29,186,848 |
| **Total Commodity Supply [1]** | 520,755 | 902,048 | 594,549 | … | 2,676,978 | 129,981 | 293,108 |  |

*[1] The Total Commodity Supply is added to this table, showing the actual total supply of the commodity in the corresponding column. This is equal to the total output of commodity* $c$ *produced by all sectors, which is the sum of all elements in the corresponding column of commodity* $c$ *in the Make table, plus other components such as imports or changes in inventories which increase the actual supply of commodity to be used in the production of industries (or consumption of final users).*

**Table A2: USE Table (2012)**

This table is extracted from the *Use* table (2012), provided by the Bureau of Economic Analysis (BEA), showing the use of 17 commodities by 15 sectors and Final users in the US. Each commodity is displayed in a row and each sector is presented in a column. The element in row $c$, column $j$ (${Use}_{cj}$) reports the value of commodity $c$ that sector (or final users) $j$ uses as the input for its production (or consumption). The sum of all elements in a row is the Total Commodity Output and the sum of all elements in a column is the Total Sector Output.

(Millions of dollars)

| **Commodities / Sectors** | AGR | MNG | … | OS | GOV | Total Inter-mediate | Personal consumption expenditures | … | Total Final Uses (GDP) | **Total Commodity Output** |
| --- | --- | --- | --- | --- | --- | --- | --- | --- | --- | --- |
| AGR | 87,000 | 96 | … | 194 | 6,053 | 386,940 | 71,294 | … | 67,522 | 454,461 |
| MNG | 2,195 | 52,843 | … | 707 | 27,507 | 715,120 | 136 | … | -147,177 | 567,943 |
| … | … | … | … | … | … | … | … | … | … | … |
| GOV | 27 | 13 | … | 1,982 | 8,173 | 94,349 | 66,274 | ... | 2,582,179 | 2,676,528 |
| Used | -34 | 756 | … | 6,186 | -33 | 38,058 | 53,945 | … | -29,516 | 8,542 |
| Others | 988 | 1,614 | … | 473 | 18,028 | 117,363 | -72,340 | … | -114,456 | 2,906 |
| **Total Intermediate** | 273,062 | 254,251 | … | 228,274 | 1,230,273 | 1,041,409 | - | ... | ... | - |
| Compensation of employees | 48,063 | 90,527 | … | 250,937 | 1,703,805 | - | - | ... | - | - |
| Taxes on production and imports, less subsidies | -1,079 | 40,302 | … | 18,984 | -22,518 | - | - | ... | - | - |
| Gross operating surplus | 130,431 | 227,969 | … | 78,070 | 478,202 | - | - | ... | - | - |
| **Total Value Added** | 179,573 | 358,798 | … | 347,991 | 2,159,489 | 16,197,010 | - | ... | 16,197,007 | - |
| **Total Sector Output** | 452,635 | 613,049 | … | 576,265 | 3,389,762 | - | 11,006,814 | … | - | 29.186,848 |

**Table A3: SHARE Table (2012)**

This table is the constructed *SHARE* table (2012), demonstrating the proportion of the commodity supplies that each sector accounts for. Each sector is presented in a row and each commodity is shown in a column. The element in row $i$*,* column $c$ (${SHARE}_{ic}$) shows the percentage of the total supply of commodity $c$ produced by sector $i$, calculated as the value of commodity $c$ produced by sector $i$ (the element in row $i$, column $c$ of the *Make* table) divided by the total supply of commodity $c$. For example, sector AGR produces 86.37% of the total supply of commodity AGR, obtained from the *Make* table as 449,781 divided by 520,755).

(%)

| Sectors / Commodities | AGR | MNG | UTL | CTN | MFG | WST | RT | TPW | INF | FIN | PRO | EH | ES | OS | GOV | Used | Others |
| --- | --- | --- | --- | --- | --- | --- | --- | --- | --- | --- | --- | --- | --- | --- | --- | --- | --- |
| AGR | 86.37 | - | - | 0.08 | 0.00 | - | - | - | - | - | - | - | 0.16 | - | - | - | - |
| MNG | - | 62.46 | - | 1.39 | 0.40 | 0.13 | - | - | - | - | 0.05 | - | - | - | - | - | - |
| UTL | - | 0.01 | 76.52 | 0.17 | - | - | 0.00 | 0.23 | - | - | 0.01 | - | - | - | 0.06 | - | - |
| CTN | - | - | - | 92.14 | - | - | - | - | - | - | 0.01 | - | - | - | - | - | - |
| MFG | - | 0.46 | - | 1.04 | 75.25 | 4.21 | - | - | - | 0.00 | 4.67 | - | - | 0.02 | - | 3.29 | - |
| WST | 0.11 | - | - | 0.20 | 0.08 | 94.63 | - | 0.19 | - | 0.19 | 0.40 | - | - | 7.87 | - | - | - |
| RT | 0.03 | - | - | 0.59 | 0.18 | - | 96.97 | 0.31 | 0.06 | 0.18 | 0.15 | 0.07 | 2.89 | 11.42 | - | - | - |
| TPW | 0.11 | - | 0.02 | 0.14 | - | 0.01 | 0.00 | 95.56 | - | 0.01 | 0.11 | 0.00 | 0.02 | 0.12 | - | - | - |
| INF | - | - | - | 0.12 | 0.02 | 0.77 | 0.14 | - | 97.26 | 0.19 | 6.35 | 0.24 | 0.00 | 0.30 | - | - | - |
| FIN | - | - | - | 1.13 | - | 0.02 | 0.09 | 0.06 | - | 97.06 | 0.25 | 0.00 | 0.00 | 0.22 | - | - | - |
| PRO | - | 0.01 | - | 0.15 | 0.04 | 0.20 | 0.42 | 0.00 | 1.41 | 0.03 | 81.00 | 0.07 | - | 0.48 | - | 0.43 | - |
| EH | - | - | - | 0.22 | - | - | 0.83 | - | - | 0.08 | 1.00 | 88.56 | 3.24 | 0.00 | - | - | - |
| ES | - | - | - | 0.23 | - | - | 0.54 | - | 0.02 | 0.08 | 0.17 | 0.07 | 88.36 | 0.37 | - | 0.10 | - |
| OS | - | - | - | 0.04 | - | 0.03 | 0.35 | - | 0.04 | 0.03 | 0.04 | 0.01 | 0.26 | 78.23 | - | - | - |
| GOV | 0.64 | 0.02 | 23.11 | 2.36 | 0.09 | - | 0.66 | 2.51 | 0.29 | 1.08 | 3.04 | 10.78 | 5.01 | 0.50 | 99.93 | 2.75 | 0.99 |

**Table A4: REVSHARE Table (2012)**

This table is the constructed *REVSHARE* table (2012), showing the value of goods traded between any pairs of sectors in the US. The element of row $i$, column $j$ (${REVSHARE}_{ij}$) demonstrates the total value of the goods flowing from sector $i$ to sector $j$ (i.e., the total value of all commodities that sector $j$ buys from sector $i$), obtained as ${\sum SHARE}_{ic}{\times Use}_{cj}$, for all commodity $c$. For example, the total value of all commodities that sector AGR buys from sector MNG is 1,780 million of dollars, obtained as the sum of the products of all elements in the MNG row of the *SHARE* table and all elements in the AGR column of the *Use* table, correspondingly.

(Millions of dollars)

| Sector | AGR | MNG | UTL | CTN | MFG | WST | RT | TPW | INF | FIN | PRO | EH | ES | OS | GOV |
| --- | --- | --- | --- | --- | --- | --- | --- | --- | --- | --- | --- | --- | --- | --- | --- |
| AGR | 75,145 | 89 | 10 | 558 | 241,812 | 1,542 | 2,764 | 124 | 59 | 180 | 2,452 | 231 | 4,390 | 179 | 5,323 |
| MNG | 1,780 | 33,403 | 22,482 | 7,921 | 372,056 | 502 | 352 | 898 | 755 | 2,108 | 1,669 | 1,166 | 1,247 | 746 | 20,011 |
| UTL | 3,497 | 7,575 | 20,363 | 2,983 | 58,925 | 12,348 | 22,641 | 12,194 | 5,372 | 49,635 | 12,143 | 17,696 | 21,532 | 3,397 | 19,107 |
| CTN | 1,573 | 4,923 | 6,787 | 195 | 14,672 | 2,047 | 3,260 | 6,026 | 2,410 | 109,958 | 1,690 | 2,246 | 2,711 | 3,554 | 72,492 |
| MFG | 64,285 | 55,437 | 22,719 | 210,273 | 1,499,117 | 61,183 | 43,368 | 122,490 | 68,314 | 62,041 | 121,755 | 145,267 | 75,949 | 43,085 | 301,493 |
| WST | 37,960 | 12,248 | 5,971 | 49,703 | 316,265 | 45,789 | 18,380 | 29,879 | 18,947 | 22,124 | 30,001 | 45,921 | 20,431 | 11,039 | 57,835 |
| RT | 1,214 | 988 | 1,544 | 60,322 | 22,882 | 4,787 | 7,393 | 14,349 | 3,132 | 13,775 | 8,703 | 6,271 | 9,737 | 6,416 | 6,177 |
| TPW | 9,831 | 12,095 | 20,690 | 16,860 | 141,339 | 71,256 | 65,344 | 106,817 | 16,057 | 26,772 | 36,729 | 19,397 | 11,333 | 4,659 | 55,528 |
| INF | 1,264 | 5,997 | 4,652 | 9,857 | 35,982 | 36,535 | 25,823 | 9,903 | 189,129 | 71,639 | 94,430 | 40,788 | 23,889 | 11,767 | 99,927 |
| FIN | 35,924 | 30,688 | 11,792 | 30,518 | 91,174 | 122,510 | 124,713 | 79,799 | 63,338 | 917,657 | 197,951 | 215,223 | 89,727 | 68,111 | 146,947 |
| PRO | 3,400 | 42,626 | 23,351 | 45,524 | 215,926 | 202,812 | 116,632 | 46,443 | 161,262 | 298,488 | 374,956 | 189,610 | 118,580 | 33,447 | 220,101 |
| EH | 118 | 606 | 625 | 1,128 | 3,384 | 4,958 | 5,834 | 1,356 | 3,478 | 6,423 | 7,484 | 32,010 | 4,031 | 3,340 | 24,647 |
| ES | 595 | 1,238 | 2,601 | 1,351 | 10,907 | 8,339 | 6,018 | 10,124 | 32,179 | 46,670 | 48,034 | 40,003 | 26,076 | 4,747 | 19,076 |
| OS | 576 | 1,038 | 534 | 4,902 | 12,749 | 18,615 | 9,364 | 13,223 | 6,356 | 22,460 | 19,007 | 20,185 | 10,331 | 6,194 | 19,869 |
| GOV | 2,572 | 4,848 | 11,264 | 4,171 | 40,899 | 34,352 | 21,103 | 16,766 | 14,397 | 62,656 | 31,788 | 29,269 | 20,788 | 6,112 | 31,458 |

**Table A5: CUST Table (2012)**

This table is the constructed *CUST* table (2012), showing the importance of the role of a sector as the customer of the other sector. The element of row $i$ and column $j$ (${CUST}_{ij}$) demonstrates the role of sector $j$ in the customer profile of sector $i$ (i.e., the proportion of the revenue of sector $i$ that is generated by sector $j$). For example, element ${CUST}_{21}$ shows that sector AGR accounts for 0.29% of the revenue of sector MNG, obtained by element ${REVSHARE}_{21}$ (1,780) divided by the total output of sector MNG in the *Make* table (613,049).

(%)

| Sector | AGR | MNG | UTL | CTN | MFG | WST | RT | TPW | INF | FIN | PRO | EH | ES | OS | GOV |
| --- | --- | --- | --- | --- | --- | --- | --- | --- | --- | --- | --- | --- | --- | --- | --- |
| AGR | 16.60 | 0.02 | 0.00 | 0.12 | 53.42 | 0.34 | 0.61 | 0.03 | 0.01 | 0.04 | 0.54 | 0.05 | 0.97 | 0.04 | 1.18 |
| MNG | 0.29 | 5.45 | 3.67 | 1.29 | 60.69 | 0.08 | 0.06 | 0.15 | 0.12 | 0.34 | 0.27 | 0.19 | 0.20 | 0.12 | 3.26 |
| UTL | 0.76 | 1.64 | 4.41 | 0.65 | 12.77 | 2.68 | 4.91 | 2.64 | 1.16 | 10.76 | 2.63 | 3.83 | 4.67 | 0.74 | 4.14 |
| CTN | 0.15 | 0.46 | 0.63 | 0.02 | 1.37 | 0.19 | 0.30 | 0.56 | 0.22 | 10.23 | 0.16 | 0.21 | 0.25 | 0.33 | 6.75 |
| MFG | 1.11 | 0.96 | 0.39 | 3.64 | 25.98 | 1.06 | 0.75 | 2.12 | 1.18 | 1.07 | 2.11 | 2.52 | 1.32 | 0.75 | 5.22 |
| WST | 2.29 | 0.74 | 0.36 | 3.00 | 19.11 | 2.77 | 1.11 | 1.81 | 1.14 | 1.34 | 1.81 | 2.77 | 1.23 | 0.67 | 3.49 |
| RT | 0.09 | 0.07 | 0.11 | 4.30 | 1.63 | 0.34 | 0.53 | 1.02 | 0.22 | 0.98 | 0.62 | 0.45 | 0.69 | 0.46 | 0.44 |
| TPW | 0.98 | 1.21 | 2.06 | 1.68 | 14.11 | 7.11 | 6.52 | 10.66 | 1.60 | 2.67 | 3.67 | 1.94 | 1.13 | 0.46 | 5.54 |
| INF | 0.09 | 0.43 | 0.34 | 0.71 | 2.60 | 2.64 | 1.87 | 0.72 | 13.68 | 5.18 | 6.83 | 2.95 | 1.73 | 0.85 | 7.23 |
| FIN | 0.71 | 0.61 | 0.23 | 0.61 | 1.81 | 2.43 | 2.48 | 1.58 | 1.26 | 18.22 | 3.93 | 4.27 | 1.78 | 1.35 | 2.92 |
| PRO | 0.11 | 1.42 | 0.78 | 1.51 | 7.18 | 6.74 | 3.88 | 1.54 | 5.36 | 9.92 | 12.46 | 6.30 | 3.94 | 1.11 | 7.31 |
| EH | 0.01 | 0.03 | 0.03 | 0.05 | 0.15 | 0.22 | 0.26 | 0.06 | 0.15 | 0.28 | 0.33 | 1.41 | 0.18 | 0.15 | 1.09 |
| ES | 0.05 | 0.11 | 0.24 | 0.12 | 1.00 | 0.76 | 0.55 | 0.93 | 2.94 | 4.27 | 4.39 | 3.66 | 2.38 | 0.43 | 1.74 |
| OS | 0.10 | 0.18 | 0.09 | 0.85 | 2.21 | 3.23 | 1.62 | 2.29 | 1.10 | 3.90 | 3.30 | 3.50 | 1.79 | 1.07 | 3.45 |
| GOV | 0.08 | 0.14 | 0.33 | 0.12 | 1.21 | 1.01 | 0.62 | 0.49 | 0.42 | 1.85 | 0.94 | 0.86 | 0.61 | 0.18 | 0.93 |

**Table A6: SUPP Table (2012)**

This table is the constructed *SUPP* table (2012), demonstrating the importance of a sector as the supplier of the other sector. The element of row $i$ and column $j$ (${SUPP}_{ij}$) shows the role of sector $i$ in the supplier profile of sector $j$ (i.e., the proportion of the total input of sector $j$ that is purchased from sector $i$). For example, element ${SUPP}_{21}$ shows that sector MNG accounts for 0.55% of the inputs of sector AGR, obtained by element ${REVSHARE}_{21}$ (1,780) divided by the total commodity inputs of sector AGR in the *Use* table (the sum of Total Intermediate 273,062 and the Compensation of employees 48,063).

(%)

| Sector | AGR | MNG | UTL | CTN | MFG | WST | RT | TPW | INF | FIN | PRO | EH | ES | OS | GOV |
| --- | --- | --- | --- | --- | --- | --- | --- | --- | --- | --- | --- | --- | --- | --- | --- |
| AGR | 23.40 | 0.03 | 0.00 | 0.06 | 5.08 | 0.14 | 0.27 | 0.02 | 0.01 | 0.01 | 0.10 | 0.01 | 0.52 | 0.04 | 0.18 |
| MNG | 0.55 | 9.69 | 8.94 | 0.89 | 7.81 | 0.05 | 0.04 | 0.11 | 0.08 | 0.08 | 0.07 | 0.06 | 0.15 | 0.16 | 0.68 |
| UTL | 1.09 | 2.20 | 8.09 | 0.34 | 1.24 | 1.11 | 2.25 | 1.52 | 0.60 | 1.97 | 0.50 | 0.89 | 2.56 | 0.71 | 0.65 |
| CTN | 0.49 | 1.43 | 2.70 | 0.02 | 0.31 | 0.18 | 0.32 | 0.75 | 0.27 | 4.36 | 0.07 | 0.11 | 0.32 | 0.74 | 2.47 |
| MFG | 20.02 | 16.08 | 9.03 | 23.70 | 31.49 | 5.49 | 4.31 | 15.22 | 7.63 | 2.46 | 5.03 | 7.33 | 9.03 | 8.99 | 10.28 |
| WST | 11.82 | 3.55 | 2.37 | 5.60 | 6.64 | 4.11 | 1.83 | 3.71 | 2.12 | 0.88 | 1.24 | 2.32 | 2.43 | 2.30 | 1.97 |
| RT | 0.38 | 0.29 | 0.61 | 6.80 | 0.48 | 0.43 | 0.74 | 1.78 | 0.35 | 0.55 | 0.36 | 0.32 | 1.16 | 1.34 | 0.21 |
| TPW | 3.06 | 3.51 | 8.22 | 1.90 | 2.97 | 6.39 | 6.50 | 13.27 | 1.79 | 1.06 | 1.52 | 0.98 | 1.35 | 0.97 | 1.89 |
| INF | 0.39 | 1.74 | 1.85 | 1.11 | 0.76 | 3.28 | 2.57 | 1.23 | 21.13 | 2.84 | 3.90 | 2.06 | 2.84 | 2.46 | 3.41 |
| FIN | 11.19 | 8.90 | 4.69 | 3.44 | 1.91 | 10.98 | 12.41 | 9.92 | 7.08 | 36.41 | 8.17 | 10.85 | 10.67 | 14.21 | 5.01 |
| PRO | 1.06 | 12.36 | 9.28 | 5.13 | 4.54 | 18.18 | 11.60 | 5.77 | 18.01 | 11.84 | 15.48 | 9.56 | 14.10 | 6.98 | 7.50 |
| EH | 0.04 | 0.18 | 0.25 | 0.13 | 0.07 | 0.44 | 0.58 | 0.17 | 0.39 | 0.25 | 0.31 | 1.61 | 0.48 | 0.70 | 0.84 |
| ES | 0.19 | 0.36 | 1.03 | 0.15 | 0.23 | 0.75 | 0.60 | 1.26 | 3.59 | 1.85 | 1.98 | 2.02 | 3.10 | 0.99 | 0.65 |
| OS | 0.18 | 0.30 | 0.21 | 0.55 | 0.27 | 1.67 | 0.93 | 1.64 | 0.71 | 0.89 | 0.78 | 1.02 | 1.23 | 1.29 | 0.68 |
| GOV | 0.80 | 1.41 | 4.48 | 0.47 | 0.86 | 3.08 | 2.10 | 2.08 | 1.61 | 2.49 | 1.31 | 1.48 | 2.47 | 1.28 | 1.07 |
